# Marine sediments microbes capable of electrode oxidation as a surrogate for lithotrophic insoluble substrate metabolism

**DOI:** 10.3389/fmicb.2014.00784

**Published:** 2015-01-14

**Authors:** Annette R. Rowe, Prithiviraj Chellamuthu, Bonita Lam, Akihiro Okamoto, Kenneth H. Nealson

**Affiliations:** ^1^Department of Earth Sciences, University of Southern California, Los AngelesLos Angeles, CA, USA; ^2^Department of Molecular and Computational Biology, University of Southern California, Los AngelesLos Angeles, CA, USA; ^3^Department Marine and Environmental Biology, University of Southern California, Los AngelesLos Angeles, CA, USA; ^4^Department of Applied Chemistry, University of TokyoTokyo, Japan

**Keywords:** electromicrobiology, geobiology, lithotrophy, sulfur oxidation, iron oxidation, Halomonas, Marinobacter, Pseudomonas

## Abstract

Little is known about the importance and/or mechanisms of biological mineral oxidation in sediments, partially due to the difficulties associated with culturing mineral-oxidizing microbes. We demonstrate that electrochemical enrichment is a feasible approach for isolation of microbes capable of gaining electrons from insoluble minerals. To this end we constructed sediment microcosms and incubated electrodes at various controlled redox potentials. Negative current production was observed in incubations and increased as redox potential decreased (tested −50 to −400 mV vs. Ag/AgCl). Electrode-associated biomass responded to the addition of nitrate and ferric iron as terminal electron acceptors in secondary sediment-free enrichments. Elemental sulfur, elemental iron and amorphous iron sulfide enrichments derived from electrode biomass demonstrated products indicative of sulfur or iron oxidation. The microbes isolated from these enrichments belong to the genera *Halomonas, Idiomarina, Marinobacter*, and *Pseudomonas* of the *Gammaproteobacteria*, and *Thalassospira* and *Thioclava* from the *Alphaproteobacteria*. Chronoamperometry data demonstrates sustained electrode oxidation from these isolates in the absence of alternate electron sources. Cyclic voltammetry demonstrated the variability in dominant electron transfer modes or interactions with electrodes (i.e., biofilm, planktonic or mediator facilitated) and the wide range of midpoint potentials observed for each microbe (from 8 to −295 mV vs. Ag/AgCl). The diversity of extracellular electron transfer mechanisms observed in one sediment and one redox condition, illustrates the potential importance and abundance of these interactions. This approach has promise for increasing our understanding the extent and diversity of microbe mineral interactions, as well as increasing the repository of microbes available for electrochemical applications.

## Introduction

Marine sediments are complex environments that can be difficult to characterize physically and biologically as they are highly heterogeneous. They can support a range of temperatures, pHs, Ehs, pressures, and concentrations of organic and inorganic metabolites over a variety of scales generating a wide range of metabolic niches (Jakobsen, 2007; Stockdale et al., 2009). This environmental variability results in an impressive diversity of microorganisms and microbial metabolisms, including metabolisms specific to a given resource niche, or requiring a specific redox interface (Nealson, 1997; Tankéré et al., 2002; Jourabchi et al., 2009). Though we can assess phylogenetic diversity, our understanding of metabolic diversity and activity is often limited in these systems to the dominant and/or well-studied processes, or often, to those few organisms that can be cultivated. However, observations of previously undetected metabolisms, such as those hidden by cycling of redox species (between element reducing and element oxidizing microbes) are amassing in many marine systems (Canfield et al., 2010; Gault et al., 2011; Holmkvist et al., 2011; Stewart et al., 2012). This coupled with the fact that many lithotrophs utilize insoluble substrates that are difficult to monitor has led to these processes being largely overlooked in environmental settings. The importance of these lithotrophic and often autotrophic reactions in marine systems and how they have been grossly under estimated is slowly coming to light (Swan et al., 2011). The main goal of this work is to utilize electrochemical methods to culture microbes capable of mineral oxidation from marine sediment where lithotrophic reactions are potentially occurring.

The unknowns surrounding lithrophic mineral oxidizers in environmental systems stem from poor representation of these microbes in culture. Characterization of their physiology is limited to a few pathways and a few systems, giving us a limited set of genetic biomarkers for these processes (Ghosh and Dam, 2009; Emerson et al., 2010; Ilbert and Bonnefoy, 2013). This, in turn, has limited our understanding of the extent of microbes (how many families, genera, organizational taxonomic units [OTUs]) performing lithotrophic reactions. Approaches to culturing microbes that can better mimic the resource niche and/or redox gradient a microbe is adapted to will help improve our understanding of these microbial metabolisms and give us a means to detect their presence in these environments. In some cases, electrode cultivation may also offer a means of applying molecular, culturing or analytical methods that are inhibited by mineral byproducts, precipitates or Fenton reactions (Rabaey et al., 2007).

Electrode microbe interactions have played an important role in understanding extracellular electron transport in mineral reducing microbes. Mechanisms of extracellular electron transport (EET), the transport of electrons to substrates external to the cell, have only been well characterized in members of the genera *Shewanella* and *Geobacter*. These include direct transfer via multiheme cytochromes (Myers and Myers, 1992), transfer along conductive nanowires (Reguera et al., 2005; Gorby et al., 2006), and transfer through soluble mediators or shuttles (Lovley et al., 1996; Newman and Kolter, 2000; Marsili et al., 2008; Von Canstein et al., 2008). To date, several of these cultures have been shown to be able to reduce electrodes as a surrogate for minerals, and depending on the potential of the electrode, can serve as a source or a sink for electrons (Gregory et al., 2004; Rosenbaum et al., 2011). It is known and has been demonstrated in many environmental systems (Rabaey and Verstraete, 2005; Lovley, 2006), and in particular marine environments (Holmes et al., 2004; Ryckelynck et al., 2005; Reimers et al., 2006; White et al., 2009), that microbes can utilize electrodes as terminal electron acceptor (TEA). What is less studied and is recently gaining attention is the potential for iron oxidizing microbes to obtain electrons from electrodes as well—using the electrode as a primary electron donor (Rosenbaum et al., 2011; Summers et al., 2013). Two recent discoveries in iron oxidizing bacteria are beginning to highlight this phenomenon. In the neutraphilic iron oxidizer *Sideryoxidans lithotrophicus* ES-1 and the phototrophic iron oxidizer *Rhodopseudomonas palustris* TIE-1, homologs to the Mtr genes in *Shewanella oneidensis* MR-1 have been shown to oxidize iron *in vitro* (Jiao and Newman, 2007; Liu et al., 2012). In *S. lithotrophicus*, there is also evidence that this gene can complement the iron reducing capacity in MR-1, suggesting a similar location, interaction with iron and potentially a similar ability to utilize an electrode (though this has not been tested) (Liu et al., 2012). Direct evidence of an iron oxidizer using an electrode as an electron source has been demonstrated in the marine iron oxidizer *Mariprofundus ferroxidans* (Summers et al., 2013) and the phototrophic iron oxidizer *Rhodopseudomonas palustris* TIE-1 (Bose et al., 2014). Notably, *M. ferroxidans* does not contain an MtrAB homolog or any other known outer membrane cytochrome protein known to be involved in EET (Ilbert and Bonnefoy, 2013). Both of these observations illustrate the potential for lithotrophic microbes to be cultivated and characterized electrochemically. Additionally, the second observation supports the high potential for novel mechanisms of extracellular electron transport in mineral oxidizing microbes.

In this work, we assess electrodes poised at electron-donating (cathodic) redox potentials as a means of enriching lithotrophic mineral oxidizing microbes from an environmental system. We successfully isolated several novel electrochemically active bacteria from marine sediments that were further electrochemically characterized giving insight into both the extent and diversity of mechanisms behind metabolisms that oxidize insoluble substrates.

## Materials and methods

### Catalina harbor sediment microcosms for primary electrochemical microbial enrichment

**Figure 1:**
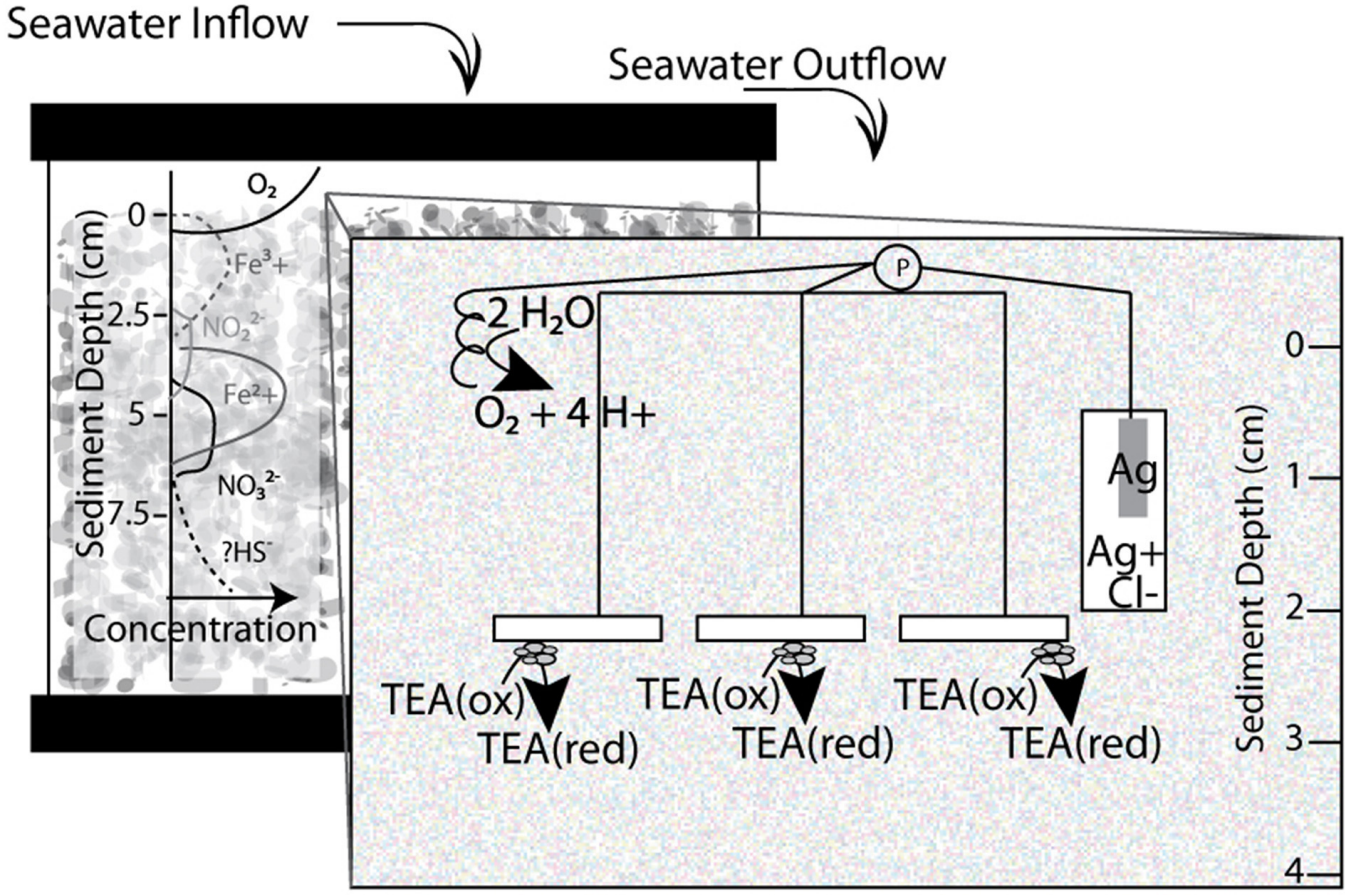
**Schematic diagram of sediment microcosms with window illustrating three electrode system incubated in 10 gallon aquaria.** Working electrodes placed between 2–4 cm in the sediment water column, while reference and counter electrodes remained in surface waters. A flow of UV treated and aerated water was added at a continuous rate to facilitate oxygen and nutrient replenishment and waste removal. P designates a potentiostat. TEA stands for terminal electron acceptor and the redox state—reduced (red) or oxidized (ox)—is indicated.

Marine sediments were collected from Catalina Harbor (33° 25.23′ N, 118° 19.42′ W; February, 2013), sieved on-site through a 1000–500 μM copper mesh, and transferred to 10 gallon aquaria (~40 L) to serve as sediment microcosms (illustrated in Figure 1). The sediments were allowed to settle for 24 h in a constant temperature room maintained between 10 and 15°C. During incubations a constant stream of aerated seawater was pumped into the surface water of aquaria at a rate of approximately five gallons (~20 L) per day. The seawater was pumped through a UV-treatment system to minimize bacterial and algal growth throughout the system to prevent clogging and ensure constant flow rates. Geochemical profiles (specifically, concentrations of oxygen, pH, redox and sulfide quantified with depth) were monitored using the Unisense microprofiling system (Unisense, Aarhus, Denmark) as described in Supplementary Information.

For electrode incubations, replicate indium tin oxide (ITO) plated glass electrodes (SPD Laboratory Inc. Hamamatsu, Japan) were placed three to four cm below the sediment surface to serve as working electrodes, for the three electrode microcosms (schematic in Figure 1). ITO, as a clear conductive material, was chosen as a material because it has properties beneficial for microbial ecology including compatibility with light and UV microscopy. Compared to other electrode materials, such as graphite, cleaner and more consistent results in voltammetry were observed with ITO electrodes (Rowe and Okamoto, personal observations). The ITO plated glass (5 mm by 10 mm) electrodes were constructed by attaching a wire to the ITO plated surface of the glass with Dotite® Silver Paint (SPI Supplies, West Chester, PA). The connection was covered with marine grade epoxy (Loctite, Henkel Co., Rocky Hill, CT) to prevent any exposure and/or electrolysis at the conductive epoxy interface. Reference (Ag/AgCl) and counter electrodes (platinum wire) were constructed in our lab and placed in the surface waters of each microcosm. Open circuit controls were performed in each incubation. Linear Sweep Voltammetry was performed on electrodes in freshly sieved sediments using a Pine potentiostat and associated Aftermath software (Pine Research Instrumentation, Durham, NC). Using an eDAQ quad channel potentiostat (eDAQ Inc., Colorado Springs, CO) with the described three electrodes voltage potential (ranging from 0 to −400 mV) was controlled at the working electrode. Applied redox conditions and current production were controlled and recorded via the eCorder eCHART software (eDAQ Inc., Colorado Springs, CO).

### Media and chemical analyses used for enrichments

An artificial saltwater base was utilized for all bioreactor and subsequent culture enrichment and isolation approaches (with the exception of isolation on enriched media, see below). Briefly, the base medium contained 342 mM NaCl, 14.8 mM MgCl_2_^*^6H_2_O, 0.1 mM CaCl_2_^*^2H_2_O, and 6.7 mM KCl. This media was designated “sulfate free” artificial saltwater media and was predominantly used for electrochemical tests like cyclic voltammetry. Sources of nitrogen, phosphorous and sulfur were added via ammonium chloride (10 mM), potassium phosphate (1 mM, pH 7.2), and sodium sulfate (1 mM). The basic media components along with the vitamin and trace mineral mixes added (listed in the Supplementary Materials and Methods) were based on the media developed by the MBL Microbial Diversity Course (Woodshole, MA). The 1 mM sulfate concentration was used for the “low sulfate” saltwater medium, and is much lower than natural seawaters (~28 mM) but is sufficient for microbial biosynthesis. Additional sulfate was added when testing sulfate as a potential terminal electron acceptor in enrichments. Bicarbonate was also added to anaerobic media at a concentration of 5 mM to support potential autotrophic growth without altering pH. All anaerobic media were maintained using traditional anaerobic techniques (Keefer et al., 1962; Miller and Wolin, 1974). Monitoring of the metabolites from enrichments was performed as described in the Supplemental Information.

### Secondary enrichments in sediment-free three electrode bioreactors

**Figure 2:**
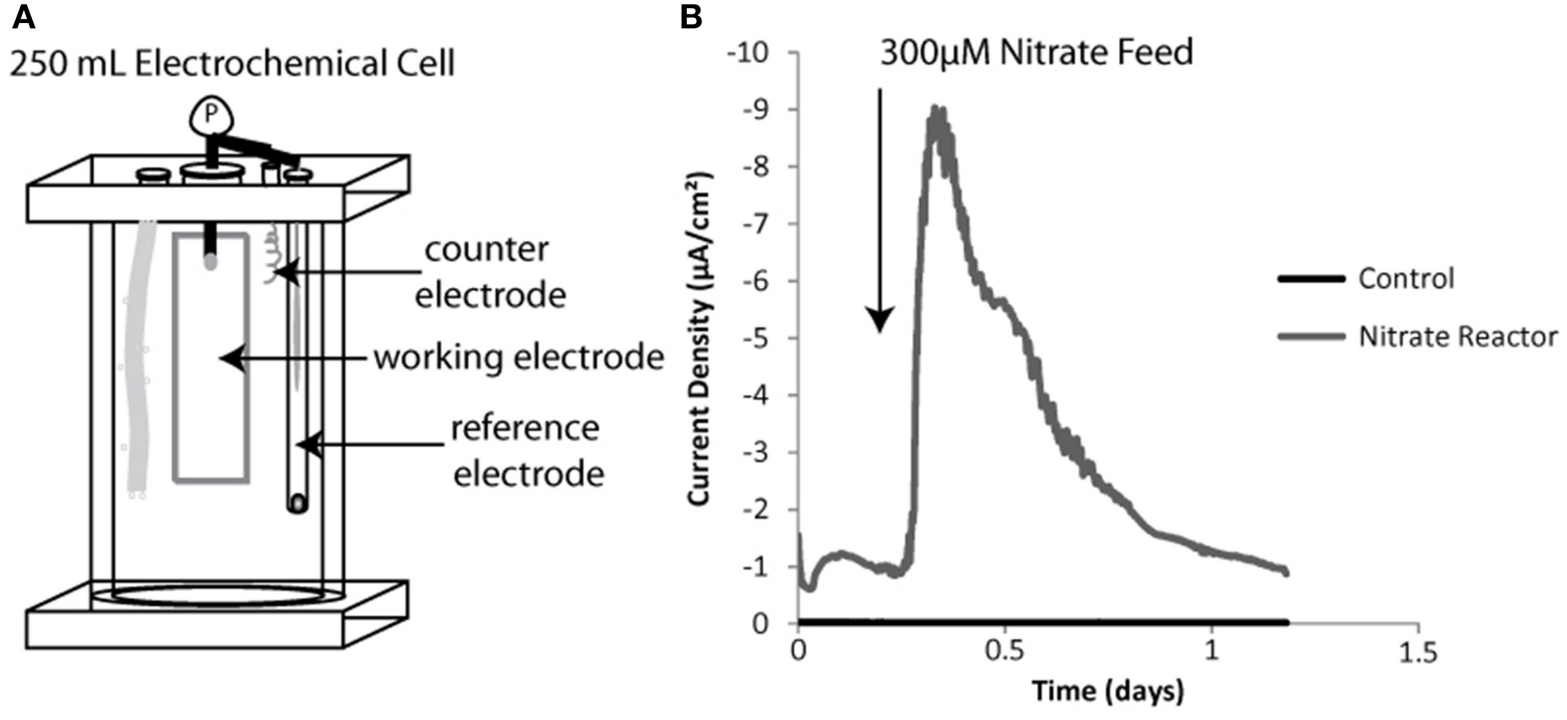
**Sediment free bioreactor schematic including three electrode system (A).** Inserted elements diagramed left to right include a gas diffuser, a working electrode, a counter electrode and a reference electrode. Example negative current production for a sediment incubated working electrode vs. a sterile or non-inoculated control both batch fed of nitrate shown in **(B)**.

At the end of sediment microcosm experiments, the working ITO electrodes were removed from sediment and placed into 0.5 L anaerobic jars filled with anaerobic low sulfate artificial saltwater media, minimizing oxygen exposure to electrode-associated microbes as much as possible. After being transferred to an anaerobic chamber, the sediment-seeded electrodes served as working electrodes in three electrode sediment-free bioreactors used for further enrichment of electrode oxidizing microbes (schematic in Figure 2A). The working electrode was poised at −400 mV (as in sediment incubations) vs. an Ag/AgCl reference electrode and platinum counter electrode in a chamber filled with up to 300 mL low sulfate artificial saltwater media (described above). Redox potential was controlled using the same eDAQ potentiostat as described above.

Bioreactors were amended with different TEAs thought to be present in the original sediment enrichments and therefore fueling electrode oxidation. Sodium nitrate, sodium sulfate, or ferric iron chloride chelated with nitrilotriacetic acid (NTA) (pH 6.5) were added at concentrations of 100–400 μM. A given bioreactor was amended with a single TEA. Current was monitored for changes in negative current production (indicative of electrode oxidation). Changes in redox state of the TEAs and/or product formation were monitored via ion chromatography (IC) (further details in the Supplementary Information) or the Ferrozine assay (Stookey, 1970).

### Tertiary microbial enrichment and isolation with insoluble electron donors

Solid substrate electron donors were chosen for further enrichment and eventual isolation of microbial strains. Reduced iron substrates, in the form of elemental iron coupons or amorphous iron sulfide (Fe_x_S_x_), were used as electron donors for iron oxidizers. No organic carbon was provided to any of the solid substrate liquid enrichments. Colloidal elemental sulfur was used as an electron donor for sulfur oxidizer enrichments (prepared as described in Byrne et al., 2009). Briefly, elemental sulfur powder was heated in water to 90°C for at least three times for an hour. Water was removed via SpeedVac (Labconco, Kansas City, MO) and approximately 100–200 mg of powdered sulfur was added to each culture. Elemental iron (Gray Cast Iron, McMaster-Carr, http://www.mcmaster.com/) was cut into 1 × 1 × 0.1 cm square chips, soaked in 1 mM HCl, rinsed and autoclaved for sterilization before adding to anaerobic saltwater media. A solution of Fe_x_S_x_ was made using equal molar ratios of sterile ferric iron sulfate and sodium sulfide as described previously (Emerson and Floyd, 2005). Nitrate, Fe(III)-NTA and sulfate served as terminal electron acceptors for solid substrate enrichments. Additions were made to balance electron donor and electron acceptor electron equivalents being supplied to the various enrichments according to the half reactions and redox potentials listed in the Supplementary Information.

Two forms of solid media containing agar were used for isolation of enriched microbes. For the majority of solid media, the base artificial saltwater media containing low sulfate was used with one of the following electron donors: Fe_2_Cl_2_, Fe(II)-NTA, S^0^, thiosulfate. Nitrate, and Fe(III)-NTA were supplied as TEAs for enrichments on these various solid substrate donors. Agar shake tubes for separating microbial isolates were created using 1–3% agar. Agar plates using 1.5% agar were used to isolate individual colonies. Difco marine broth (Difco, Lawrence, KS), an enriched marine media, was used to culture organisms capable of using organic carbon as a primary electron donor, after a series of enrichments on inorganic electron donors.

### DNA extraction from enrichments and isolates

DNA was extracted from biomass pellets from sediment free bioreactors and solid substrate electron donor enrichments (combination planktonic and scraped electrode biofilm, or cell suspension centrifuged at >13,000 × g) using the MoBio PowerSoil kit (MoBio, Carlsbad, CA) according to manufacturer's protocols. Enrichment culture volume of 2–5 mL was sampled in the anaerobic chamber, depending on enrichment. DNA extractions for sequencing of isolated microbial strains were performed on pure culture cell pellets grown overnight in DM media using the UltraClean Microbial DNA Isolation kit (MoBio, Carlsbad, CA) according to manufacturer's protocol. DNA quality and quantity was determined via absorbance spectroscopy using a Nanodrop (Thermofisher Scientific, Wilmington, DE). Samples submitted for 16S rRNA-tagged amplicon sequencing (described below) contained DNA concentrations of greater than 10 ng/ul and 280/260 ratios of greater than 1.8. Notably control (open circuit) electrodes did not generate sufficient biomass for quantification and/or sequencing.

### Sequencing and analysis of enrichments and isolates

DNA samples extracted from culture enrichments were sequenced using 16S rRNA tagged amplicon pyrosequencing by MR-DNA (Molecular Research LP, Shallowater, TX). In short, the approach amplified ribosomal sequences with a 30 cycle PCR reaction with a 53°C annealing temp using barcoded 27F universal Eubacterial primer paired with 1100R (described in Dowd et al., 2008). Amplicons were pooled, purified with Agencourt Ampure beads (Agencourt Bioscience Corporation, Beverly, MA), and sequenced using a Roche 454 FLX titanium instrument according to manufacturer protocols. Average sequence lengths of 350 bp and >3000 sequences were obtained for all samples. Quality checking (base ambiguities, homopolymers >6 bp etc.), sequence denoising (Reeder and Knight, 2010), chimera checks (Edgar et al., 2011) and bar code removal algorithms were applied using the Qiime software (Quantitative Insights into Microbial Ecology) (Caporaso et al., 2010b). OTU picking of distinct sequence types (based on 97% similarity), involved clustering of similar sequences (Edgar, 2010), sequence alignment (Caporaso et al., 2010a) and phylogenetic analysis using the green genes database (Werner et al., 2012), was also performed in Qiime using the aforementioned additional programs.

Ribosomal 16S sequences were obtained for all isolates using direct 16S rRNA amplification from pure culture DNA extracts. The universal bacterial primers 27F (5′-AGAGTTTGATCCTGGCTCAG) and 1492R (5′-GGTTACCTTGTTACGACTT) were used. Approximately 20–40 ng of PCR product from each isolate were purified with a DNA Clean Concentrator Kit (ZymoResearch, Irvine, CA), and Sanger sequencing was performed via Genewiz (La Jolla, CA) or Beckman Coulter Genomics (Danvers, MA). These nearly full length sequences were quality checked and assembled using Geneious 7.1^©^ (Biomatters, New Zealand). Alignment of sequences against the Silva database was performed using the SINA aligner (v 1.2.11) (Pruesse et al., 2012; Quast et al., 2013). Nearest cultured representative microbes were also obtained through the Silva database (Quast et al., 2013). Maximum-likelihood estimation trees were constructed from alignments of sequences and nearest neighbors using RaxML (v.8) (Stamatakis, 2014). All full length sequences have been deposited to Genbank (Accession numbers KM088025-KM088033).

### Electrochemical analysis of isolated strains

Electrochemical tests were performed on isolated strains in a three electrode electrochemical cell with a 6–10 mL capacity (described previously, Nakamura et al., 2009). An ITO plated glass electrode was used for the working electrode; the system also utilized platinum counter and Ag/AgCl reference electrodes. Chronoamperometry was performed on isolated cultures using the eDAQ quad channel potentiostat and associated software as described for previous enrichments (see sediment and bioreactor enrichments). Isolate cultures were grown on either enriched marine broth (Difco, Lawrence, KS), or saltwater base with Fe^2+^-NTA or thiosulfate as an electron donor. Both conditions consisted of either nitrate or oxygen as a TEA. Cultures grown in enriched media were added to the electrochemical cell and given 24 h to attach to an electrode charged between 100 and −100 mV. At the end of 24 h the liquid phase spent media was removed and replaced with fresh sterile low sulfate saltwater media and a −400 mV voltage potential was applied. For thiosulfate and ferrous iron cultures, cells were centrifuged anaerobically to remove media with respiration by-products. This cell suspension was then added to electrochemical cells poised at −400 mV. If nitrate was supplied as a donor incremental batch feeds of 100–400 μM nitrate were added to electrochemical cells incubated in an anaerobic chamber. Electrochemical cells amended with oxygen as a terminal electron acceptor were maintained at room temperature on the bench top with a continuous flow of air or nitrogen depending on the experimental condition. Current production was monitored over the course of these experiments and killed controls (autoclaved cell biomass) were used to test for non-biological current generation. A non-electrochemically active *Streptococcus mutans* strain (provided by Jeff McClean, JCVI) was used as a control for background oxygen reduction/negative current production (Cournet et al., 2010).

Post chronoamperometry experiments, cyclic voltammetry (CV) was performed on the biofilms of colonized electrodes, spent media from electrochemical cells containing planktonic biomass and electrochemical cell filtrates to look at the electrochemical activity or responses of each component. A Gamry Reference 600 potentiostat (Gamry Instruments, Warminster, PA) and the proprietary software Gamry Framework were used for each electrochemical assay. Parameters for CV analysis included scan rates of 5–20 mV/s over a range of −600 to 600 mV or −800 to 800 mV vs. Ag/AgCl. Gamry Echem Analyst (Gamry, Warminster, PA) was used for post run analysis of electrochemical using predominantly non-turnover conditions (in the absences of a terminal electron acceptor). Calculation of mid-point potentials, average of oxidizing and reducing peak pairs, was performed using the Echem Analyst software. Scanning Electron Microscopy was performed on electrodes from these experiments as described in Supplementary Information.

## Results

### Current production in primary (sediment microcosm) electrochemical enrichments

Catalina Harbor sediment microcosms generated cathodic/negative currents (indicative of electrons being removed from the working electrode) when applied with sufficiently negative redox potentials (Table 1). The applied voltage potentials tested in microcosms ranged from −50 to −400 mV vs. Ag/AgCl (Table 1). The most positive voltage tested (−50 mV vs. Ag/AgCl) was chosen based on the lowest redox potential measured in the sediment depth range the electrodes were placed (Figure S1). No observable differences in the measured geochemical gradients of the microcosms were detected. Specifically, oxygen and redox profiles generated similar trends across depth (Figure S1). No sulfide was detected in microcosms (likely due to the high detection limit [~10 μM total sulfide] at pH 8 (Jeroschewski et al., 1996; Revsbech, 2005) and scavenging of sulfide by iron) and the pH remained constant with depth. As the poised voltage decreased (potential became more reducing) current generation increased; the maximum average currents observed across microcosms was for electrodes poised at -400 mV (Table 1). Linear sweep voltammetry (LSV) performed in the sediment microcosms prior to microbial enrichments demonstrated that abiotic current production is minimal over the voltage range at which the incubations occurred (Figure S2). These results indicate that within the range of cathodic potentials tested, current production attributable to abiotic electrochemical reactions remains less than 100 pA/cm^2^. In addition, no exogenous terminal electron acceptors were supplied to these enrichments suggesting that naturally occurring TEAs were supporting electrode oxidation by the microbial population enriched by the electrodes. To determine the metabolic capabilities of the microbes enriched in the sediment microcosms secondary and tertiary enrichments were employed.

**Table 1:** **Current production in sediment microcosms incubated at different voltages**.

**Applied voltage (mV) vs. Ag Ag/CL**	**Duration (Days)**	**Average current (μA) ± standard deviation^#^**			
		**Microcosm 1**	**Microcosm 2**	**Microcosm 3**	**Microcosm 4**
−50	8	^*^	NT	NT	5.1 ± 3
−100	8	NT	10.2 ± 2	10.4 ± 3	NT
−300	8	−6.6 ± 3.1	−75.9 ± 14.2	−0.01 ± 0.03	−29.7 ± 19.7
−400	14	−31.1 ± 2.5	−44.9 ± 15	−27.8 ± 22.5	−64.5 ± 14.9

# *Standard deviations based on average data with outlying data points (resulting from electrical surges and shorts) removed*.

* *Indicate a lack of statistical significance in results*.

*NT indicates an experimental condition not tested*.

### Identifying TEAs in secondary enrichments of electrochemically active microbes

Relationships between electrode-oxidizing microbes and potential terminal electron accepting processes were determined using sediment-free bioreactors (Figure 2A). Bioreactors contained 300 mL liquid volume of sterile low sulfate saltwater media. Each three electrode sediment-free bioreactor was seeded with biomass by using a working electrode from the primary sediment enrichments. Batch addition of a single TEA and responses in current production were monitored relative to the control (sterile) electrode (Figure 2A). Ferric iron-NTA, nitrate and sulfate were chosen as TEAs for these experiments based on a thermodynamically favorable redox difference between the electrode and terminal electron acceptors. In addition, each of the TEAs has been detected in the native sediments, in either current (Figure S1) or previous work quantifying these compounds (Bertics and Ziebis, 2009). Oxygen was not used as a TEA in these secondary enrichments as the electrodes in the sediment enrichment were buried several centimeters below where the oxygen level fell below detection (detection limit <1 μM O_2_) (Revsbech, 2005).

Nitrate addition resulted in an increase in negative current in the sediment-free bioreactor (Figure 2B). The integrated current accounted for 22 percent of all the electrons added if each nitrate was converted to nitrogen gas (5 electrons per nitrate) and 55 percent of the electrons added if nitrite was the final reduction product (2 electrons per nitrate). The efficiency of this process was likely limited both by the use of hydrogen (naturally present in the anaerobic chamber) as an electron source for certain denitrifying populations and by the escape of gaseous intermediates. Over time current generated per batch nitrate feed decreased (data not shown), likely due to competition between electrode oxidation and hydrogen oxidation as the source of electrons for nitrate reduction. Once this was observed, the nitrate bioreactor was halted and used to seed solid substrate enrichments.

Bioreactors amended with iron or sulfate did not demonstrate clear-cut relationships between current production and addition of TEA. No significant current was generated above background as a result of sulfate addition (Figure S3). In iron amended reactors, addition of iron caused a slow initial increase in current production that never dropped to the pre-amendment baseline (Figure S3). Because of this, integrated current calculations showed far more current is generated than predicted from iron additions. The most logical explanation for this phenomenon is that oxidation processes on the counter electrode are driving re-oxidation of iron which can in turn be used by electrode oxidizing microbes and reduced again resulting in high stable current generation.

### Metabolic and phylogenetic analysis of tertiary solid substrate enrichments

As nitrate was the most successful TEA tested in the sediment-free bioreactors for electrode oxidizing microbes planktonic and attached biomass from the nitrate amended reactor was used in tertiary enrichments on various insoluble primary electron donors (PED). Solid substrates, including elemental iron, amorphous iron sulfide, and elemental sulfur were chosen as probable primary electron donors for native electrode-oxidizers enriched from Catalina sediments. No organic carbon was provided to any of the enrichments. Lithotrophic respirations were measured by the production of end-products (i.e., sulfate from sulfur or Fe^3+^ from Fe^0^ or Fe_x_S_x_). Metabolism of substrates was confirmed using two to three transfers of activity without the addition of carbon in any form other than bicarbonate (Supplemental Table S2). Not all intermediates from each pathway could be quantified (i.e., sulfite, thiosulfate, tetrathionate, and gaseous denitrification products) limiting our confirmation of the extent or specific pathways of oxidation.

**Figure 3:**
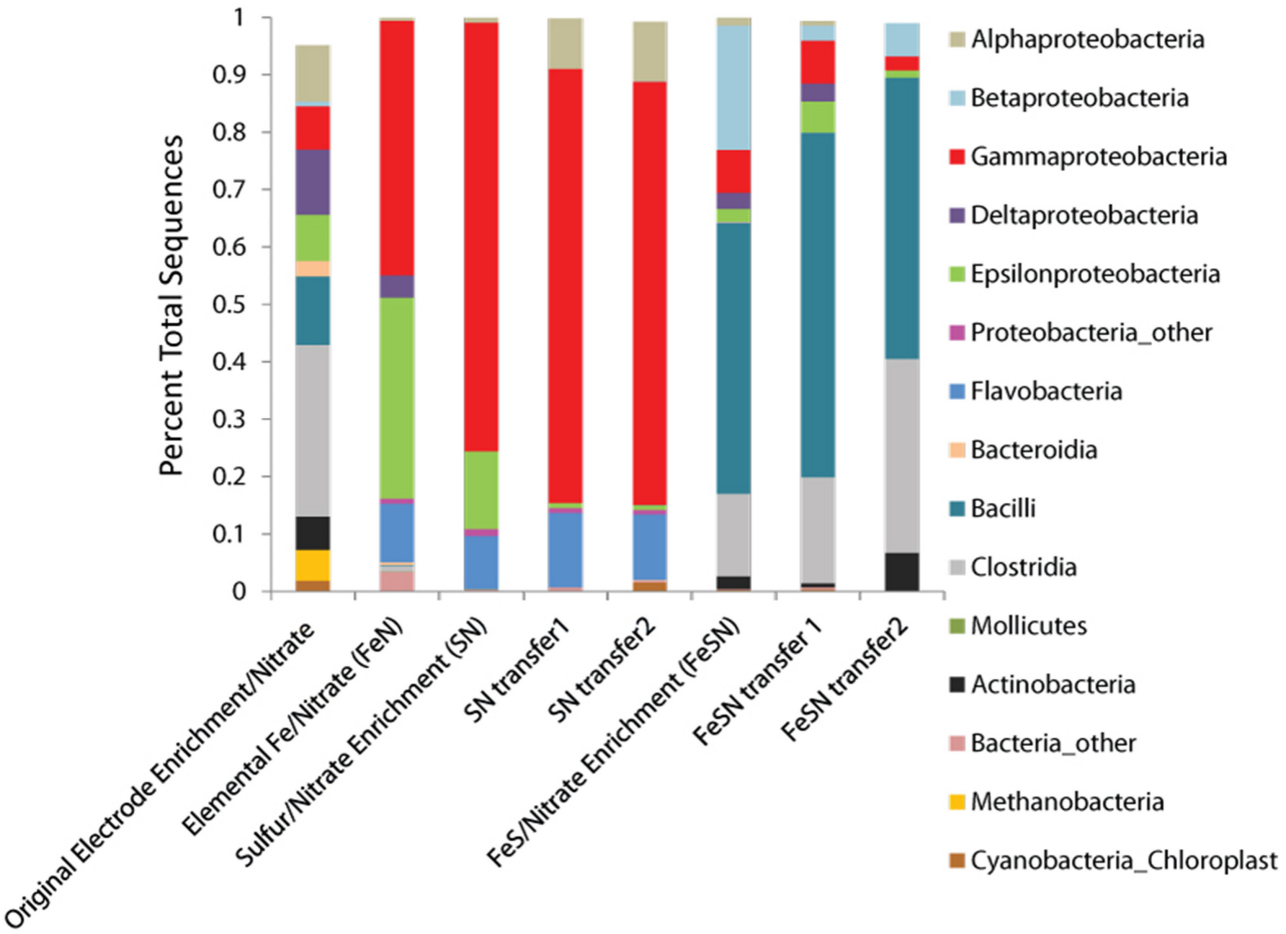
**Phyla observed in solid substrate enrichments using nitrate as a terminal electron acceptor.** The phyla recovered from the original biomass used for inoculation of cultures is designated original electrode enrichment/nitrate. Phyla were determined from 16S rRNA tagged pyrosequencing analysis of DNA extracts from enrichments, analyzed using Qiime (see Materials and Methods).

Community analysis performed using phylogenetic 16S rRNA analysis in Qiime demonstrated a phylogenetically distinct group of microbes that differed from the initial electrode community was enriched depending on the insoluble electron donor used (Figure 3, Table 2). Elemental sulfur and nitrate cultures enriched predominantly for members of the *Gamma*- and *Alphaproteobacteria*, whereas amorphous iron sulfide enrichments amended with nitrate enriched strongly for members of the *Firmicutes*. Interestingly, elemental iron enrichments looked similar in phylogenetic composition to sulfur oxidizing nitrate enrichments. Of the specific OTUs enriched on various substrates (listed in Table 2), most fall into either poorly- or un-cultivated groups or those whose representatives are not known to be electrochemically active. A few of the groups represented contain other members that are capable of sulfur or iron oxidation, such as the *Halomonas* and *Arcobacter*, though these groups are in general metabolically versatile. Members of the *Pseudomonas* are an exception to this trend in that they have been extensively cultivated and shown to be either electrochemically active (Sharma and Kundu, 2010; Su et al., 2012b) or capable of sulfur or iron oxidation, though to our knowledge not both (Sorokin, 2003; Smith et al., 2011).

**Table 2:** **Top 12 OTUS detected and abundances in tagged pyrosequencing analysis of DNA extracts from solid substrate electron donor enrichments**.

**Primary electron donor**	**Terminal electron acceptor**	**Phylum**	**OUT designation^#^**	**Percent abundance of OTU**		
**Elemental Sulfur**	**Nitrate**			**Original Enrichment**	**Transfer 1**	**Transfer 2**
		*Gammaproteobacteria*	*Gammaproteobacteria*	9	15	24
		*Gammaproteobacteria*	*Pseudomonas sp.*	45	42	21
		*Gammaproteobacteria*	*Halomonas sp.*	4	3	15
		*Flavobacteria*	*Aequorivita*	9	13	11
		*Alphaproteobacteria*	*Rhodobacteraceae*	1	8	7
		*Gammaproteobacteria*	*Pseudomonadaceae*	6	9	6
		*Gammaproteobacteria*	*Pseudoidomarina*	0	2	2
		*Gammaproteobacteria*	*Idiomarina*	6	1	2
		*Alphaproteobacteria*	*Thalassospira*	0	0	2
		*Gammaproteobacteria*	*Pseudomonadaceae*	3	2	1
		*Gammaproteobacteria*	*Marinobacter*	0	1	1
		*Epsilonproteobacteria*	*Arcobacter*	13	1	1
**Elemental Iron**	**Nitrate**			**Original Enrichment**	**NA**	**NA**
		*Gammaproteobacteria*	*Halomonas*	35		
		*Epsilonproteobacteria*	*Arcobactor*	32		
		*Flavobacteriia*	*Aequorivita*	10		
		*Unknown*	*other*	3		
		*Deltaproteobacteria*	*Pelobacteraceae*	3		
		*Gammaproteobacteria*	*Pseudoidiomarina*	3		
		*Gammaproteobacteria*	*Gammproteobacteria*	2		
		*Epsilonproteobacteria*	*Sulfurimonas*	2		
		*Gammaproteobacteria*	*Pseudomonas*	1		
		*unknownProteobacteria*	*Proteobacteria*	1		
		*Gammaproteobacteria*	*Idiomarina*	1		
		*Gammaproteobacteria*	*Halomonadaceae*	1		
**Fe_x_S_x_**	**Nitrate**			**Original Enrichment**	**Transfer 1**	**Transfer 2**
		*Firmicutes*	*Bacillus sp.*	37	47	42
		*Firmicutes*	*Clostridium sp.*	7	4	25
		*Firmicutes*	*Veillonellaceae*	0	10	7
		*Actinobacteria*	*Propionibacterium sp.*	1	1	7
		*Firmicutes*	*Paenibacillus sp.*	7	7	4
		*Betaproteobacteria*	*Rhodocyclaceae*	8	0	3
		*Firmicutes*	*Anoxybacillus sp.*	0	0	2
		*Gammaproteobacteria*	*Acinetobactor sp.*	4	1	2
		*Betaproteobacteria*	*Diaphorobacter sp.*	1	2	2
		*Firmicutes*	*Lachnospiraceae*	0	0	2
		*Epsilonproteobacteria*	*Sulfurospirillum sp.*	2	3	1
		*Firmicutes*	*Staphylococcus sp.*	2	3	1

# *OTU designation based on percent similarity to known sequences (determined using Qiime, see Materials and Methods)*.

*NA designates not applicable because transfers were not performed or not sequenced most likely due to inactivity*.

### Isolation of electrochemically active facultative lithotrophs

**Figure 4:**
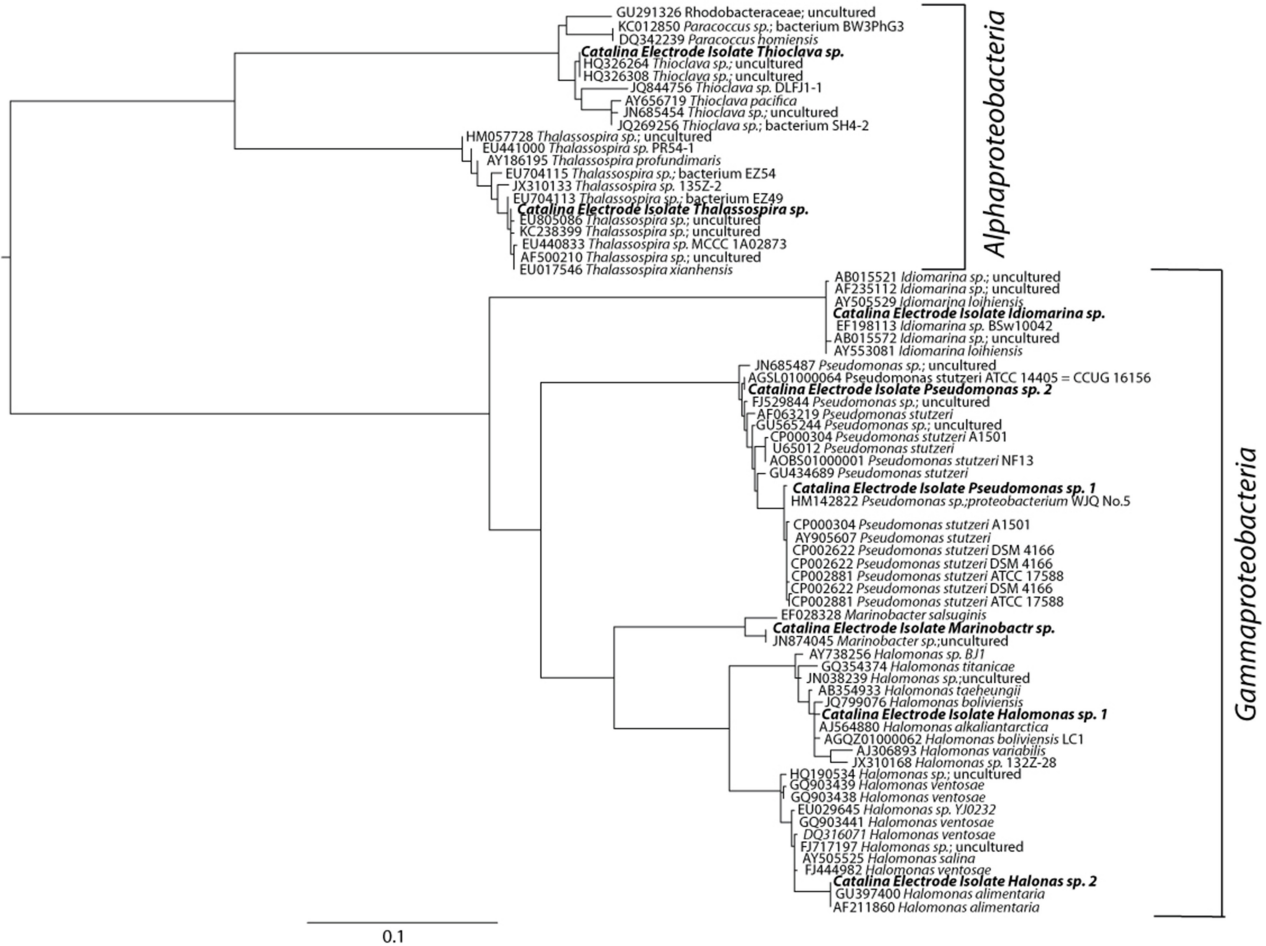
**Maximum likelihood estimation phylogenetic tree based on 900 bp of the 16 rRNA gene.** sequences aligned using the SINA aligner. Nearest neighbors obtained from the Green Genes database (accession numbers indicated). The scale bar indicates nucleotide substitutions computed using RaxML. The *Thalassospira sp*. (HM057728) was designated as the tree root.

Isolates from the most promising enrichments (elemental iron and sulfur oxidation coupled to nitrate reduction) were among the dominant members enriched on the corresponding solid substrates; isolates observed in the top 10 sequences returned from elemental iron and sulfur solid substrate enrichments (Table 2, Figure 4). Putative sulfur oxidizers were isolated via anaerobic plating with thiosulfate and nitrate. The majority of isolates altered the pH of the surrounding media, indicative of acid production (traditional thiosulfate oxidation to sulfate), or base production (incomplete oxidation of thiosulfate to tetrathionate, Gijs Kuenin personal communication, Sorokin, 2003). From thiosulfate plates, multiple isolates were obtained including putative tetrathionate producers from the *Oceanospirillales (Halomonas* genus), *Pseudomonadales (Pseudomonas* genus) and *Alteromonadales (Idiomarina* genus) orders of the *Gammaproteobacteria* (Figure 4). *Alphaproteobacteria* from the *Thalassospira* and *Thioclava* genera were also isolated (Figure 4). While, many of the microbes isolated in this work are phylogenetically related to metabolically versatile organisms and contain representatives capable of iron and sulfur oxidation, the cultured representatives from the *Idiomarina* and *Thalassospira* have only been shown to utilize reduced carbon as an electron source. Though no carbon was provided to enrichments, it is possible that reduced carbon could be acquired from agar containing isolation methods. It is suspected that all isolates are capable of facultative heterotrophy in that pure culture growth has been observed in minimal saltwater media lacking reduced carbon as well as enriched marine media.

The iron-oxidizing microbes that were isolated fell within four genera: *Halomonas*, *Marinobacter*, *Pseudomonas* and *Idiomarina*. These isolates were obtained through a combination of plating on a complex marine media, and isolation via agar shake tubes containing nitrate and FeCl_2_ or ferrous iron NTA. Notably, several strains of *Halomonas* and *Pseudomonas* were isolated from all iron enrichments. *Marinobacter* isolates were only obtained from enrichments containing FeS and *Idiomarina* strains were only isolated from elemental iron enrichments. In addition to all isolates being capable of heterotrophy, all isolates obtained were capable of growth with oxygen as a terminal electron acceptor.

**Figure 5:**
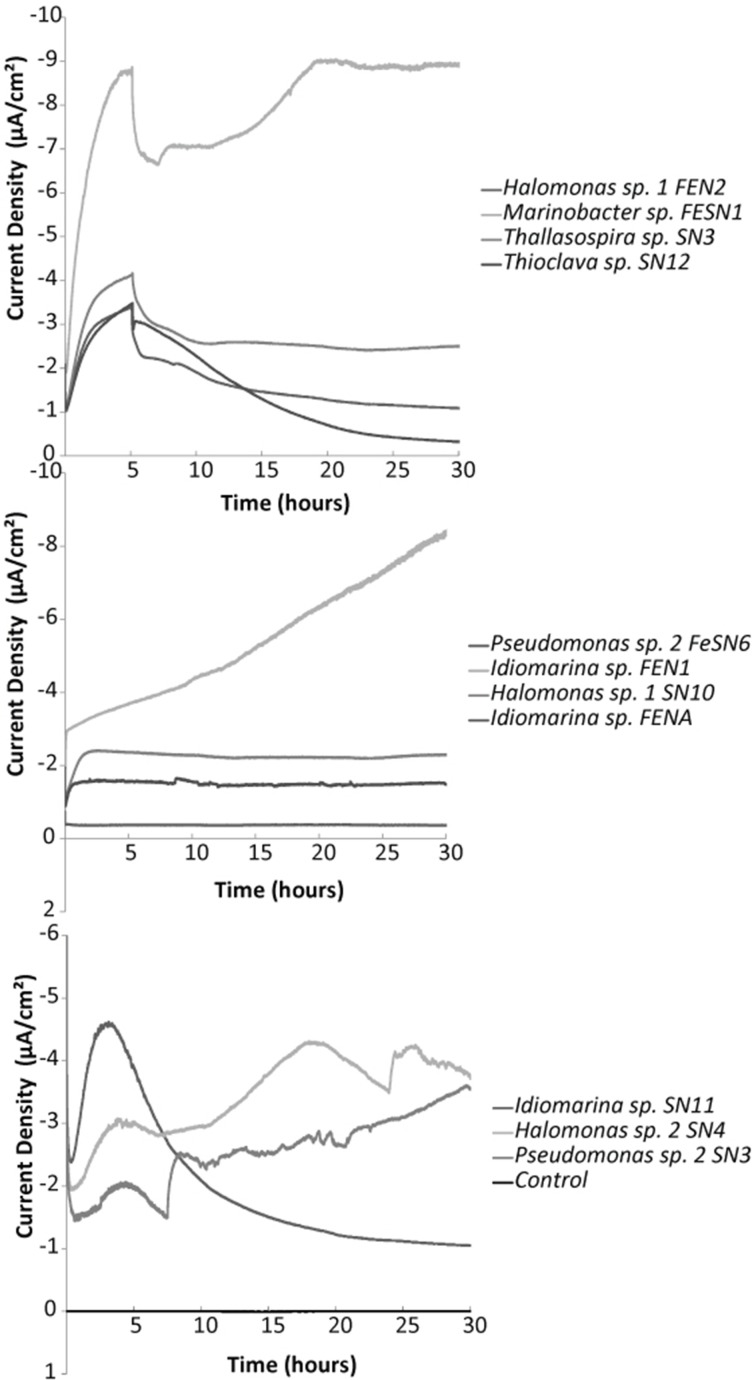
**Representative chronoamperometry profiles for oxygen fed electrochemical cells with various electrode oxidizing isolates compared to a control (non-electrochemically active) *Streptococcus mutans* strain.** Electrodes were poised at −400 mV vs. Ag/AgCl. Graphs illustrate the first 30 h post inoculation with biomass.

All isolates were tested with chronoamperometry and shown to be electrochemically active or capable of electrode oxidation with nitrate or oxygen as a terminal electron acceptor (examples in Figure 5, Figure S4). Killed controls demonstrated no response to addition of nitrate (Figure S4). Though the isolates tested demonstrated electrochemical activity with nitrate (increase in negative current production coinciding with nitrate addition [Supplementary Figure S4]) high current densities were difficult to obtain. For example coulombic efficiency of electron capture from the electrode to nitrate varied between 0.5 and 5%. This was likely due to competition between hydrogen and the electrode as an electron source as well as the potential production of toxic/growth inhibiting intermediates as coulombic efficiency often dropped over time. As the vast majority of denitrifying bacteria also use oxygen, more extensive electrochemical studies were performed with oxygen as a TEA.

Chronoamperometry with oxygen also revealed significant increases in negative current production compared to abiotic and non-electrogenic bacterial controls (examples in Figure 5, Figures S5, S6). Electrode oxidation could be tied to the presence of oxygen, as current dramatically decreased to near background levels when pure nitrogen was provided to the electrochemical cells (Figure S5). Long term incubations of several of the strains exhibited increased current production over time (2–4 weeks in the electrochemical cell without the addition of an exogenous carbon substrate) (Figure S6). This suggests autotrophic growth of certain strains as no organic carbon was added to any of the electrochemical cells. However, this affect and the strength of response (observed current densities) varied across the different strains.

### Variation in mechanisms of electron transport across isolated strains

**Figure 6:**
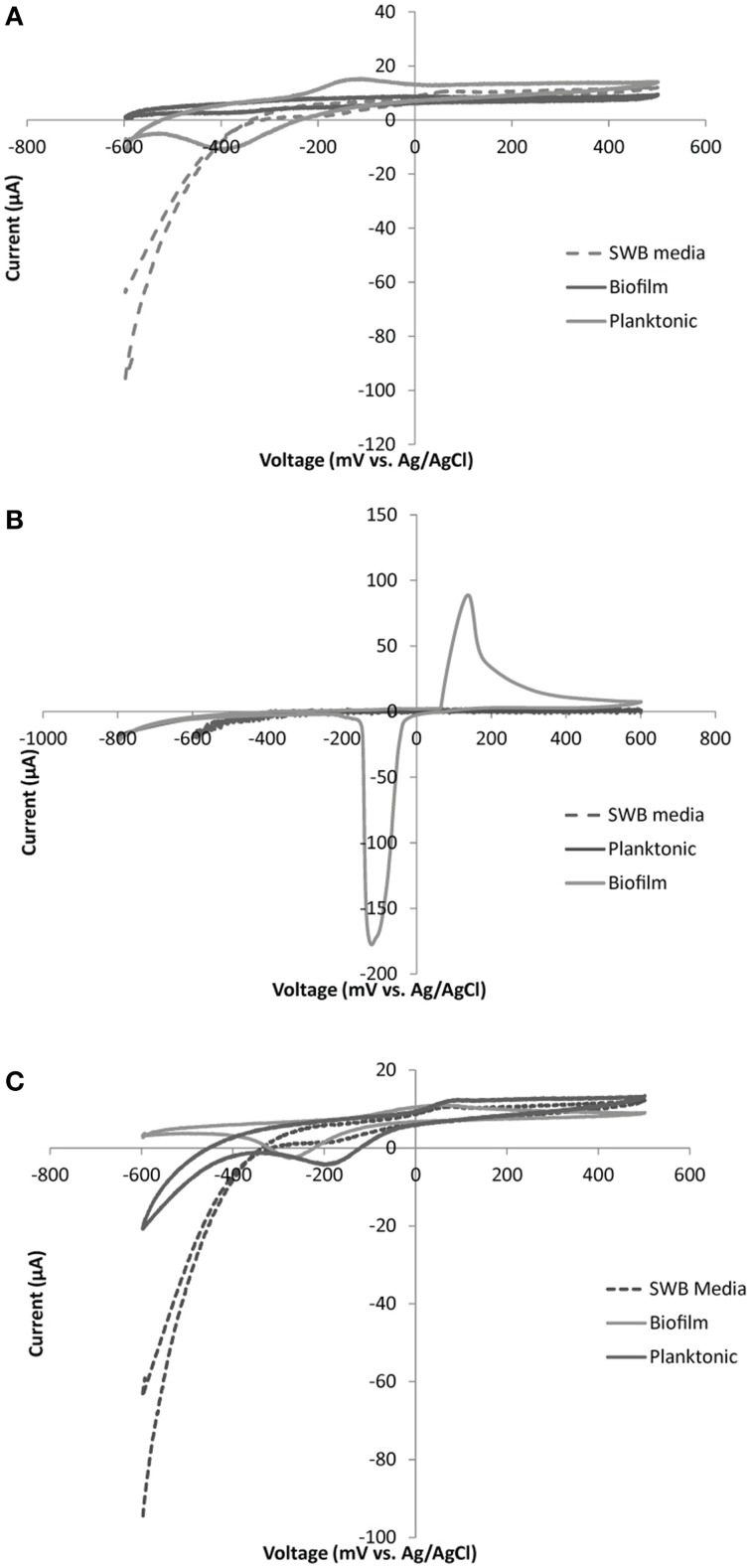
**Representative CV plots for: (A) *Marinobacter sp*. FeSN3, (B) *Idiomarina sp*. SN11 and (C) *Thallassospira sp*. SN3.** Plots are displayed for control (SWB media), planktonic cells (suspended biomass removed from electrochemical cell), and biofilm (electrode attached biomass) samples for each organism. Each CV was run with a scan rate of 5 mV/s over a voltage range from −600 to +500 mV with the exception of the biofilm sample from *Idiomarina sp*. SN11 **(B)** which was run from −800 to +600 mV.

Different electrochemical patterns were discerned among strains isolated and shown to be electrochemically active via chronoamperometry (Table 3). SEM imaging (Supplement Figure S7) and cyclic voltammetry (Figure 6, Figure S8) were used on different cellular populations or fractions (biofilm, planktonic, and cell free/filtered media) to help elucidate the type of mechanisms involved in electrode oxidation. Biofilm formation/physical attachment to the electrodes was confirmed via SEM (Figure S7). We also investigated the electrochemical signals (peaks indicative of oxidation or reduction in cyclic voltammetry) of attached vs. planktonic cells, and spent media to look for the presence of electrochemically active mediators (representative examples in Figure 6, and Figure S8). We noted variation in whether cell generate predominant electrochemical signals is in the planktonic phase (Figure 6A), biofilm phase (Figure 6B), or both phases (Figure 6C). Notably, no concreate indication of redox-active mediators was observed. Redox activity in one or more of these areas allowed for calculation of a midpoint potential for a given electrochemically active feature (i.e., outer membrane protein) (Table 3). The midpoint (MP) potential between observed oxidation and reduction during cyclic voltammetry (CV) varied across isolates and strains. In general, more reduced MP potentials were observed in iron-oxidizing isolates (−86 to −294 mV range for iron oxidizers compared to 8 to −121 mV for sulfur oxidizing isolates) (Table 3). This trend held across strains from the same genera that were isolated on different substrates suggesting different electron transport mechanisms even across strains. Attachment of microbes to electrodes appeared to be the dominant mode of electrode interaction, though a few cell types made poor/no discernable biofilms. Specifically, *Halomonas* strain FeN2 had visible cell aggregation near the electrode surface that were readily removed with the electrochemical cell supernatant.

**Table 3:** **Electrochemical characterization of isolates after chronoamperometric measurements with nitrate or oxygen as a terminal electron acceptor in either planktonic (suspended cells from electrochemical reactors) or biofilm (attached to incubated electrode) associated biomass**.

**Species cluster**	**Strain name**	**ED from enrichment**	**EA used for chronoamperometry**	**Measured midpoint potential (mV vs. Ag/AgCl)**	**Form of electrochemical activity (MP detected)**
*Pseudomonas sp. 1*	SN 8	Elemental sulfur	Nitrate	−65.7 ± 12	Biofilm
	SN 9	Elemental sulfur	Nitrate	−104 ± 3.5	Biofilm
*Pseudomonas sp. 2*	SN 5	Elemental sulfur	Oxygen	−43.2 ± 6.8	Biofilm, Planktonic(±)
	FeN 3	Elemental iron	Nitrate	−258 ± 34	Biofilm
*Halomonas sp. 1*	FeN 1	Elemental iron	Oxygen	−294 ± 5.6^*^	Biofilm, Planktonic^*^
	FeN 2	Elemental iron	Nitrate	−248 ± 6.5	Planktonic, Biofilm(±)
*Halomonas sp. 2*	SN 4	Elemental sulfur	Oxygen	−21.5 ± 19.7, 86 ± 32	Biofilm
	SN 10	Elemental sulfur	Nitrate	−108 ± 3.8	Biofilm
	FeSN 2	Iron sulfide	Oxygen	−258 ± 34	Biofilm
*Marinobacter sp.*	FeSN 1	Iron sulfide	Nitrate	−176.2 ± 30	Biofilm
	FeSN 3	Iron sulfide	Nitrate	−184 ± 24.9	Biofilm, Planktonic(±)
*Idiomarina sp.*	SN 11	Elemental sulfur	Oxygen	7.9 ± 0.8	Biofilm
	FeNA	Elemental iron	Oxygen	−86^#^	Biofilm
*Thalassospira sp.*	SN 1	Elemental sulfur	Nitrate	−103.7 ± 3.2, 51.8 ± 9	Biofilm, Planktonic
	SN 3	Elemental sulfur	Nitrate	−121.4 ± 3.2, 48.5 ± 9	Biofilm, Planktonic
*Thioclava sp.*	SN 12	Elemental sulfur	Oxygen	−54.4^#^	Biofilm(±)

* *Measurement taken from planktonic data*.

# *Only one replicate obtained*.

(±) *Indicates weak but quantifiable signal*.

## Discussion

Catalina Harbor sediment, like most sediment, contains a gradient of redox conditions. Work from Bertics et al., analyzed sediment cores taken from Catalina Harbor and showed that invertebrate burrowing activities generated a diversity of redox conditions by distributing oxygen (resulting in the production of oxidized ferric iron) and potentially nitrate and sulfate to lower sediment depths (Bertics and Ziebis, 2009). This activity creates a wide suite of redox niches with gradients of multiple electron donor and electron acceptor (oxygen, nitrate, ferric iron, etc.) couples and is likely responsible for the extensive microbial diversity and heterogeneity of OTU distributions observed (Bertics and Ziebis, 2009). The lower sediment depths were dominated by ferrous iron compared to the upper sediments, ranging from 400 to 1600 μmol per cm^3^ sediment (Bertics and Ziebis, 2009). The high iron content of these sediments allows for the possible presence of marine neutraphilic iron-oxidizing bacteria and also results in scavenging of sulfide generated from sulfate reduction. Notably, neither previous work (Bertics and Ziebis, 2009), nor our work demonstrated detectable hydrogen sulfide. However, over time, the production of black precipitates in sediment microcosms was observed suggesting active sulfate reduction (observations by A. Rowe and B. Lam). This work also suggests the potential importance/abundance of elemental sulfur produced from tetrathionate and sulfide in this system in addition to reduced FeS. Though elemental sulfur is rarely quantified in marine sediments, it has been shown to be the most abundant sulfur intermediate present in both Black Sea and North Sea sediments (1.0–0.1 μmole/cm^3^) (Zopfi et al., 2004). This could have important implications on sediment nutrient cycling.

In order to enrich for microbes capable of oxidizing the insoluble inorganic substrates present in Catalina Harbor, we used an electrode to impose a constant redox state and mimic a solid substrate or mineral surface, predicting that mineral-oxidizing microbes would be capable of taking electrons from an electrode. We tested a range of electron donating redox potentials to demonstrate the utility of this approach for enriching a notoriously difficult to culture group of microbes. Cathodic currents were sustained consistently at redox potentials lower than −300 mV vs. Ag/AgCl, which is significant in that these potentials are energetically capable of donating electrons to many oxidized redox couples (oxygen, nitrate, etc.) but do not cause significant hydrogen formation (empirical observations Figure S2, and Gregory et al., 2004). The ability of microbes to utilize cathodes has been demonstrated previously (Clauwaert et al., 2007a,b; Jia et al., 2008; Butler et al., 2010; Cournet et al., 2010; Desloover et al., 2011; Hsu et al., 2012; Su et al., 2012a). This capability has been identified in predominantly mineral reducing microbes (Rabaey et al., 2007; Rosenbaum et al., 2011). While other works have shown the feasibility of using electrodes at electron accepting redox potentials for enriching metal or sulfur reducing bacteria from marine sediments (Holmes et al., 2004; Reimers et al., 2006; White et al., 2009), only a few studies have looked at microbial communities on cathodes and predominantly from applied systems (wastewater, fuel cells etc.) (Rabaey et al., 2008; Faimali et al., 2010; Wrighton et al., 2010; Strycharz-Glaven et al., 2013). Isolation of microbes from cathode enrichments capable of oxygen reduction using electrons obtained from an electrode yielded members of the *Betaproteobacteria, Gammaproteobacteria*, and *Bacteriodetes* (Rabaey et al., 2008) or *Actinobacteria* and *Flavobacteria* (Erable et al., 2010). In neither group of isolates was the ability to use an insoluble substrate shown as these strains were grown heterotrophically and/or using hydrogen as the primary electron donor (Rabaey et al., 2008; Erable et al., 2010).

In sediment-free secondary enrichments electrode oxidation was coupled to the reduction of the anaerobic TEA amendments (nitrate and ferric iron). However, we observed no evidence of coupled sulfate reduction and electrode oxidation. This could be due to the unfavorable difference in redox potential between sulfate (E^0^ = −590 mV vs. SHE at pH 8.0, see Supplementary Information) and oxidation at the poised working electrode (−203 mV vs. SHE). Though it is difficult to predict the exact energetic gain under the proposed conditions, it seems likely that the energy gained from this reaction would be insufficient to effectively compete with other electrode oxidizing processes. This will be tested in future work by testing lower redox potentials. Alternatively, electrode oxidizing sulfate reducers may be more sensitive to oxygen then the other microbes tested in this work and so were inhibited in sediment-free reactors due to counter electrode processes (potential for oxidation of water to oxygen).

Tertiary enrichments focused on nitrate as a TEA couple for mineral oxidizing metabolisms. Multiple solid substrate donors, including elemental iron, elemental sulfur and reduced (amorphous) FeS, were shown to enrich for a variety of different phylogenies. A distinct shift in microbial groups being enriched was observed for the different iron substrates (elemental iron vs. reduced FeS). Interestingly microbial communities enriched on elemental iron, shared a similar phylogenetic composition to the elemental sulfur enrichments; these enrichments were dominated by *Gammaproteobacteria*. The similarity between the elemental iron and sulfur enrichments could be due to charge similarity (0) between these compounds compared with ferrous iron (+2) enrichments which were dominated by members of the *Firmicutes*. Nonetheless many of the same genera were isolated from these different enrichments. Interestingly, members of the *Marinobacter* were only isolated from enrichments containing Fe^2+^ or FeS. Though FeS is an amorphous and insoluble substrate it is known to release small amounts of Fe^2+^ a substrate potentially amenable to different oxidation processes and thus targeting different microbial groups. Alternatively these differential enrichment patterns could also be a result of a stronger selection for organisms that utilize insoluble rather than soluble substrates. The selection of only certain groups with these particular electron donor/electron acceptor couples suggest that there are even more physiologies enriched by the electrodes than accounted for in isolation methods. Future work will address looking at a greater range of solid substrate enrichments and/or electrochemical isolation techniques to target other electrochemically active populations.

Given the preliminary enrichment of microbes based on electrochemical treatment, it was not surprising that all of the microbes isolated from solid substrate enrichments demonstrated electrochemical activity. Though variation was noted across strains in terms of current densities observed, this variation could be a function of the ecology of the organisms. In essence, the rate of electron uptake per cell is likely related to the max reaction rates (V_max_) for the overall reaction which could vary across strains. Notably the current densities observed for the strains isolated fall within the range of current densities observed in other organism isolated from cathodes. Specifically, Erable et al., isolated strains with current densities of 0.02–1μA/cm^2^ (Erable et al., 2010) and Rabaey et al., observed maximum current densities of 27–69 μA/cm^2^ for various isolates (Rabaey et al., 2008). In this system current densities were increased with the length of time the organisms were incubated in the electrochemical cells (Figure S6). This observation suggests the ability of at least some of the strains isolated to grow autotrophically using an electrode as the sole electron donor. Additionally that lack of carbon substrates available could limit the current production in strains that were unable to grow using only the electrode. Further studies are required to better address the optimization of electrochemical activity in these organisms for applied purposes.

To our knowledge this is the first instance of an electrochemically active sulfur oxidizing microbe, though several sulfate reducing microbes have been shown to be capable of cathode oxidation. Likewise, members of the *Pseudomonas* genera have previously been shown to be electrochemically active and some *Pseudomonas* strains are capable of sulfur oxidation. The electrochemical activity of sulfur oxidizers may be linked to the ability of microbes to use extracellular elemental sulfur. However, the majority of biochemical work on sulfur oxidizers demonstrates the use of elemental sulfur intracellularly (Friedrich et al., 2005)—most proteins characterized in sulfur oxidation are cytoplasmic with the exception of the phototrophic sulfur oxidizer *Allochromatium vinosum*. However, it is likely that the organisms isolated on elemental sulfur do not use the traditional pathways of sulfur oxidation and/or all of the intermediates. This is further supported by the observation that many sulfur oxidizing *Gammaproteobacteria* often do not contain genes from the canonical sulfur oxidizing pathway (Sox), supporting a potentially novel biochemistry in addition to potentially novel EET mechanisms.

The majority of isolates obtained from elemental sulfur enrichments are likely to oxidize thiosulfate to tetrathionate given the pH increase observed on thiosulfate plates (thiosulfate being the sole electron donor) in contrast to acid production with oxidation to sulfate. Notable this metabolism has been observed previously in member of the *Halomonas* and *Pseudomonas* (Sorokin, 2003). Metabolically this product results in one less electron obtained for sulfur compounds compared with oxidation to sulfate. Additionally, tetrathionate is a “dead end” product in that it cannot be further microbially metabolized (Sorokin, 2003). However, tetrathionate in the presence of sulfide can abiotically be re-reduced, regenerating thiosulfate and generating elemental sulfur from sulfide. As both of these are substrates readily oxidized by tetrathionate producers this particular sulfur oxidation pathway could have ecological advantages to traditional sulfur oxidation. It has been postulated that this pathway could allow for energy storage in the form of elemental sulfur (Sorokin, 2003). This may be especially important in unstable or low nutrient environments. Alternatively, elemental sulfur may be the preferred substrate utilized by these sulfur oxidizers.

Differences in electrochemical activity across isolated strains, even from the same genera, were observed in this work. This extends not only to the portion of electrons removed from the electrode but also to the predicted mode of electrode interaction (i.e., biofilm vs. planktonic) and the dominant redox potential of the proteins interacting with the electrode (i.e., midpoint potential). In the *Halomonas* isolates, we observed organisms that generated thickly populated and sparsely populated biofilms (Figure S5). We also observed the formation of suspended aggregated cells that only loosely attached to the electrode and may not have survived SEM processing (e.g., *Marinobacter sp*. Figure S5C). This may speak to an intermediate mode of attachment; a mode that is more transient or more porous than a traditional biofilm. We observed no conclusive evidence of the presence of soluble electrochemically active compounds (potential mediators) in the spent media (where cells were removed) though our preliminary CV evidence in some *Pseudomonas* strains display patterns that suggest mediator binding. However, more sensitive methods may be required to detect such interactions.

The observations that the microbes isolated in this work contain multiple metabolisms (i.e., organisms that can oxidize both iron and sulfur), and perform the same metabolism using different physiological mechanisms (predicted from the variation in midpoint potentials of dominant redox active proteins), suggest that the single redox condition applied can enrich a diversity of microbes. Interestingly, the diversity of midpoint potentials suggests that variable energetic recoveries and efficiencies may be colonizing electrodes under a given redox condition. There is the potential for a tradeoff between the amount of energy recovered and the efficiency of a given redox interaction between a soluble substrate and a microbial EET pathway. Which microbes do better under the instituted redox conditions, and or how altering the redox conditions alters the community will be the focus of future work.

Given the data obtained from this work, electrochemical enrichment of microbes from environmental systems is a promising approach to: (1) expand the range of different microbes in culture collections, (2) better understand the importance of redox conditions and microbe mineral interactions in the microbial ecology of a system, and (3) expand the microbes available for electrochemical applications (e.g., biocathodes in microbial fuel cells). Many of these isolates are commonly observed in marine sediments in 16S rRNA gene surveys and in the subsurface (Ivanova et al., 2000; Inagaki et al., 2003; Kaye, 2004; Kaye et al., 2011; Smith et al., 2011; Dong et al., 2013; Kato et al., 2013) suggesting that that these oxidative metabolisms may be widespread though not generally identified. Identification of genes involved in these processes or extending these enrichment techniques to other environments could help inform the extent of these processes in nature. It should be noted that electromicrobiology, though a young field, has been demonstrating its utility in application including alternative energy generation (microbial fuel cells), removal of water contaminants such as heavy metal, chlorinated compounds, or nitrate from water (bioremediation), energy storage and the generation of small organic molecules (electrosynthesis) (He and Angenent, 2006; Rabaey et al., 2007). Electrochemical cultivation methods will add to the repository of microbes capable of electrode interactions, and may help maximize the efficiency or productivity of these applications.

### Conflict of interest statement

The authors declare that the research was conducted in the absence of any commercial or financial relationships that could be construed as a potential conflict of interest.

## Abbreviations

TEA: Terminal electron acceptor
ITO: indium tin oxide
NTA: nitrilotriacetic acid
EET: extracellular electron transfer
OTU: organizational taxonomic unit
IC: ion chromatography
SEM: scanning electron microscopy
CV: cyclic voltammetry
LSV: linear sweep voltammetry.
