# Supplementary material for: Marine sediments microbes capable of electrode oxidation as a surrogate for lithotrophic insoluble substrate metabolism
Source: Front Microbiol. 2015 Jan 14;5:784. doi: 10.3389/fmicb.2014.00784 (PMC4294203; doi:10.3389/fmicb.2014.00784)
Supplement: Supplementary file 1 [file Presentation1.PDF]

Supplementary Information For:

Marine sediments microbes capable of electrode oxidation as a surrogate for lithotrophic insoluble substrate metabolism

Annette R. Rowe, Prithiviraj Chellamuthu, Bonita Lam, Akihiro Okamoto, Kenneth Nealson

Supplementary Materials and Methods

Sediment Microprofiling

Sediment mircroprofiling was performed using Unisense micrelectrodes and the micromanipulator (Aarhus, Denmark). A custom attachment for the micromanipulator was constructed to allow attachment to sediment microcosms—maximizing the sediment depths profiled. Microelectrodes were calibrated and used according to the manufacturer's protocols (Unisense, Aarhus, Denmark). Profiles were performed in the sediment water column over a distance of approximately six cm. Profiles were taken in microcosms both prior to addition of electrodes and a weekly basis during electrode incubations.

Media Supplements

Trace mineral mix added the following components at designated concentrations: $FeSO_4$ (7.5 µM), H_3BO_4 (48 µM), $MnCl_2$ (0.5 µM), $CoCl_2$ (6.8 µM), $NiCl_2$ (1 µM), $CuCl_2$ (12 nM), $ZnSO_4$ (0.5 µM), Na_2MoO_4 (0.15 µM), $NaVO_3$ (2 µM), Na_2WO_4 (75 nM), Na_2SeO_3 (23 nM), HCl (20 µM). The vitamin mix added a final concentration of 0.1 µg/ml of the following components, unless different concentration designated, to media: riboflavin, biotin (0.03 µg/ml), thiamine HCl, L-ascorbic acid, d-Ca-pantothenate, folic acid, nicotinic acid, 4-aminobenzoic acid, pyridoxine HCl, lipoic acid, NAD, thiamine pyrophosphate, cyanocobalamin (0.01 µg/ml). All solutions were made up at 1000x concentration

Metabolite Analysis

Chemical analysis of soluble redox forms of terminal electron acceptors (chelated ferric iron, nitrate, sulfate), metabolic intermediates (nitrite), and soluble end products of oxidized electron donors (sulfate, chelated ferrous iron) was performed using ion chromatography (IC) and/or spectrophotometric assays. Anion analysis via a Metrohm Ion Chromatograph (Metrohm, Riverview, FL) was performed using an anions separation column (Metrosep A SUPP 250, Metrohm) designed for seawater samples with 3.2 mM sodium bicarbonate 1 mM sodium carbonate running buffer containing 2% acetonitrile as per manufacturer protocol (flow rate 0.7 mL/min). Spectrophotometric assays to quantify iron (Ferrous and Total, Ferrozine Assay) (Stookey, 1970) and sulfide (Cline Assay) (Cline, 1969) were previously described.

Reduction Oxidation Potential Calculations

Standard redox potentials were calculated using the following relationship for pH 0 and corrected for pH 8:

$$\Delta E^\circ = \frac{\Delta G^\circ}{-nF}.$$

Standard free energies (25°C, 1 atm, 1 M both products and reactants including or excluding protons depending on pH) were calculated for each reaction as listed above using the SUPCRT92 software (Johnson *et al.*, 1992), with thermodynamic data taken from (Shulte *et al.*, 2001; Shock *et al.*, 1997; Shock and Helgeson, 1988).

The following redox potentials for the given half reactions were calculated:

Electron Acceptors	E_0 (V vs. SHE) pH 0	$E_0^{'}$ (V vs. SHE) pH 8
$O_2 + 4\ e^- + 4\ H^+ \rightarrow 2\ H_2O$	1.23	0.77
$NO_3^- + 2\ e^- + 3\ H^+ \rightarrow NO_2^- + H_2O$	0.82	0.34
$NO_2^- + e^- + 2H^+ \rightarrow NO + H_2O$	1.06	0.11
$2\ NO + 2\ e^- + H^+ \rightarrow N_2O + H_2O$	1.7	1.22
$N_2O + 2\ e^- + H^+ \rightarrow N_2 + H_2O$	1.72	1.24
$Fe^{3+} + e^- \rightarrow Fe^{2+}$	0.77	0.77
$SO_4^{2-} + 2\ e^- + 2H^+ \rightarrow SO_3^{2-} + H_2O$	-0.11	-0.59
$2\ SO_3^{2-} + 4\ e^- + 6H^+ \rightarrow S_2SO_3^{2-} + 3\ H_2O$	0.68	-0.04
$S_2SO_3^{2-} + 4\ e^- + 6H^+ \rightarrow 2\ S^0 + 3\ H_2O$	0.49	-0.23
$S^0 + 2\ e^- + 1H^+ \rightarrow HS^-$	0.06	0.11
Electron Donors		
$2\ S^0 + 3\ H_2O \rightarrow S_2SO_3^{2-} + 4\ e^- + 6H^+$	-0.49	0.23
$2\ S_2SO_3^{2-} \rightarrow S_4O_6^{2-} + 2\ e^-$	-0.02	-0.02
$S_2SO_3^{2-} + 3\ H_2O \rightarrow 2\ SO_3^{2-} + 4\ e^- + 6H^+$	-0.68	0.04
$SO_3^{2-} + H_2O \rightarrow SO_4^{2-} + 2\ e^- + 2H^+$	0.11	0.59
$Fe^0 \rightarrow Fe^{2+} + 2\ e^-$	0.47	0.47
$Fe^{2+} \rightarrow Fe^{3+} + 1\ e^-$	-0.77	-0.77

Scanning Electron Microscopy

ITO electrodes were removed from the electrochemical cells and immediately fixed with 2% glutaraldehyde in basal saltwater media. Electrodes were stored in the glutaraldehyde solution at 4°C until dehydration. Dehydration was performed as described in Weber et al., (Weber, Jr. *et al.*, 1978). In brief,

the fixed samples were washed with basal media and subjected to a series of ethanol washes of increasing concentration with final dehydration step using hexamethyldisilazane (HMDS). Post dehydration, samples were sputter coated with carbon as described in (Hsu *et al.*, 2012). Scanning Electron microscopy was performed using a JEOL 7100 SEM (University of Southern California Microscopy Facility).

References

Cline, J. D. (1969). Spectrophotomic Determination of Hydrogen Sulfide in Natural Waters. *Limnol. Oceanogr.* 14, 454–458.

Hsu, L., Masuda, S. a., Nealson, K. H., and Pirbazari, M. (2012). Evaluation of microbial fuel cell Shewanella biocathodes for treatment of chromate contamination. *RSC Adv.* 2, 5844. doi:10.1039/c2ra20478a.

Johnson, J. W., Oelkers, E. H., and Helgeson, H. C. (1992). SUPCRT92: A software package for calculating the standard molal thermodynamic properties of minerals, gases, aqueous species, and reactions from 1 to 5000 bar and 0 to 1000°C. *Comput. Geosci.* 18, 899–947. doi:10.1016/0098-3004(92)90029-Q.

Shock, E. L., and Helgeson, H. C. (1988). Calculation of the thermodynamic and transport properties of aqueous species at high pressures and temperatures: Correlation algorithms for ionic species and equation of state predictions to 5 kb and 1000°C. *Geochim. Cosmochim. Acta* 52, 2009–2036. doi:10.1016/0016-7037(88)90181-0.

Shock, E. L., Sassani, D. C., Willis, M., and Sverjensky, D. a (1997). Inorganic species in geologic fluids: correlations among standard molal thermodynamic properties of aqueous ions and hydroxide complexes. *Geochim. Cosmochim. Acta* 61, 907–50. Available at: http://www.ncbi.nlm.nih.gov/pubmed/11541225.

Shulte, M. D., Shock, E. L., and Wood, R. H. (2001). The temperature dependence of the standard-state thermodynamic properties of aqueous nonelectrolytes. *Geochim. Cosmochim. Acta* 65, 3919–3930.

Stookey, L. L. (1970). Ferrozine---a new spectrophotometric reagent for iron. *Anal. Chem.* 42, 779–781. doi:10.1021/ac60289a016.

Weber, Jr., W., Pirbazari, M., and Melson, G. (1978). Biological growth on activated carbon: an investigation by scanning electron microscopy. *Environ. Sci. Technol.* 12, 817–819. doi:10.1021/es60143a005.

Supplemental Table S1. Sequences used for tagged sequencing of 16S rRNA genes from the following samples (see Materials and Methods for details).		
**Sample ID**	**Barcode Sequence**	**Linker Primer Sequence**
Original Nitrate Electrode Enrichment	AGACGAGT	AGRGTTTGATCMTGGCTCAG
Elemental Iron/Nitrate Enrichment	AGACTGTC	AGRGTTTGATCMTGGCTCAG
Elemental Sulfur/Nitrate (SN) Enrichment	AGACCACT	AGRGTTTGATCMTGGCTCAG
SN transfer 1	AGACCAGA	AGRGTTTGATCMTGGCTCAG
SN transfer 2	AGACCTCA	AGRGTTTGATCMTGGCTCAG
Iron Sulfide/Nitrate (FeSN) Enrichment	AGACTCAC	AGRGTTTGATCMTGGCTCAG
FeSN transfer 1	AGACTCTG	AGRGTTTGATCMTGGCTCAG
FeSN transfer 2	AGACTGAG	AGRGTTTGATCMTGGCTCAG

Supplemental Table S2. Metabolite analysis of replicate solid substrate enrichment cultures (1-3), controls (C) and transfer cultures (data for first transfer only shown [A]). Enrichments were seeded with biomass from sediment free electrochemical reactors. Iron quantification was performed via Ferrozine assay and nitrate/nitrite analysis was performed via ion chromatography (described in Supplementary Materials and Methods).

Electron Donor (ED)	Electron Acceptor (EA)	Oxidation of ED (i.e., Sulfate or Fe^{3+} production relative to control)	Reduction of EA (i.e., Nitrate consumption relative to control)
Elemental Sulfur (S_o)<br>Primary Enrichment (second 10 mM nitrate feed at 18 days)	Nitrate	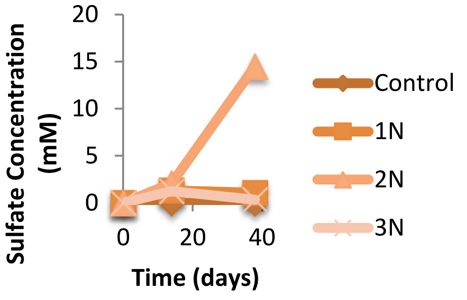	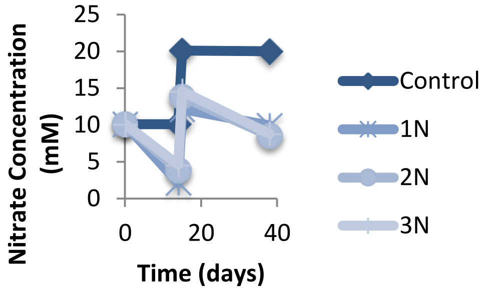
Elemental Sulfur (S_o)<br>First Transfer (second 10 mM nitrate feed at 18 days)	Nitrate	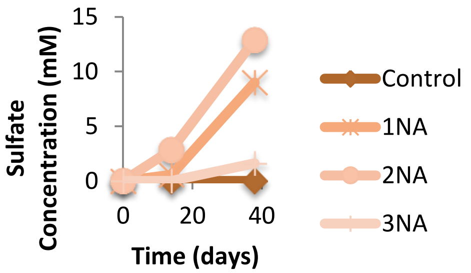	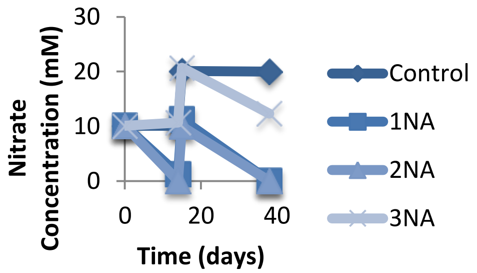
Reduced Iron-sulfide (Fe_xS_x)<br>Primary Enrichment (second 15 mM feed at 14 days)	Nitrate	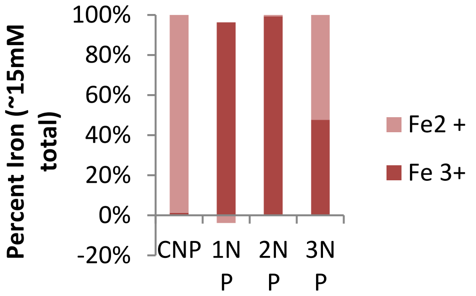	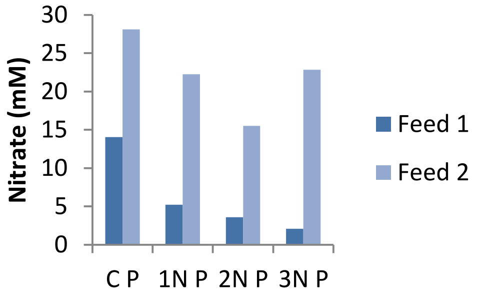
Reduced Iron-sulfide (Fe_xS_x)<br>First Transfer (second 15 mM feed at 14 days-)	Nitrate	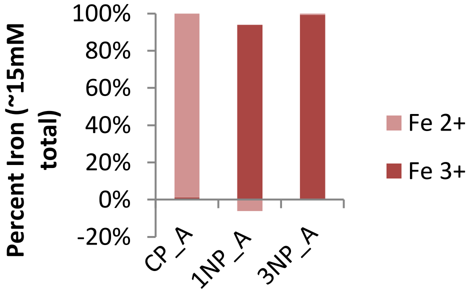	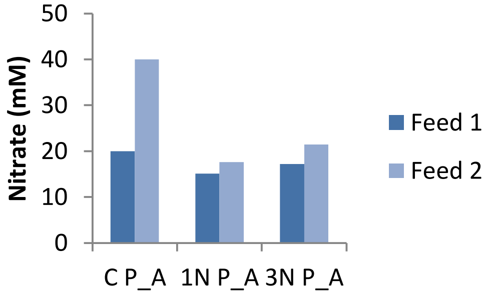

<table>
<tr><td>Elemental Iron (Fe_o)</td><td>Nitrate (N)</td><td rowspan="2">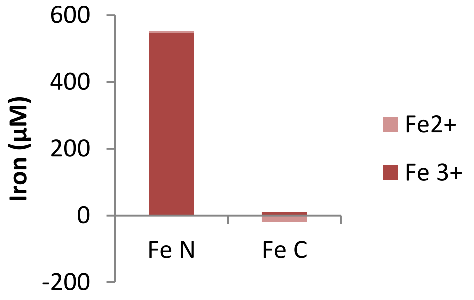
</td><td rowspan="2">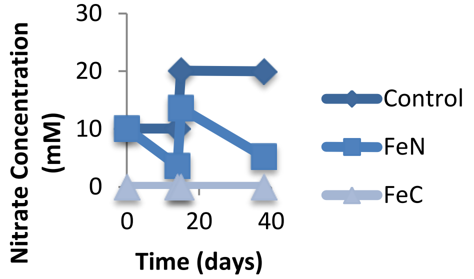
</td></tr>
<tr><td colspan="2">Primary Enrichment (second 10 mM nitrate feed at 18 days)</td></tr>
</table>

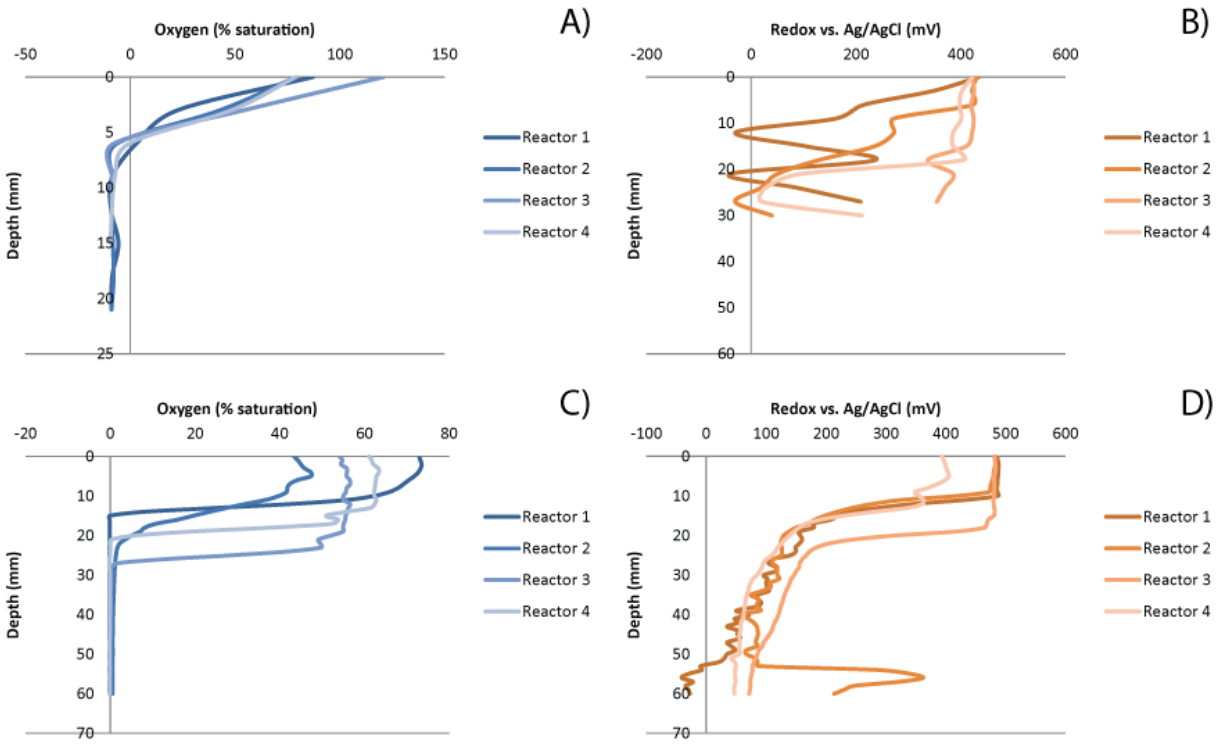

Figure S1. Example oxygen (A&C) and Redox (B&D) microprofiles from marine sediment microcosms displayed. Quadruplicate reactors incubated with electrodes and various potentials. Initial (A&B) and Final (C&D) profiles are illustrated. Initial profiles are shorter due to depth constraints on micromanipulator. Modification of microprofiling equipment eliminated this limitation for later profiles (See Supplementary Materials and Methods).

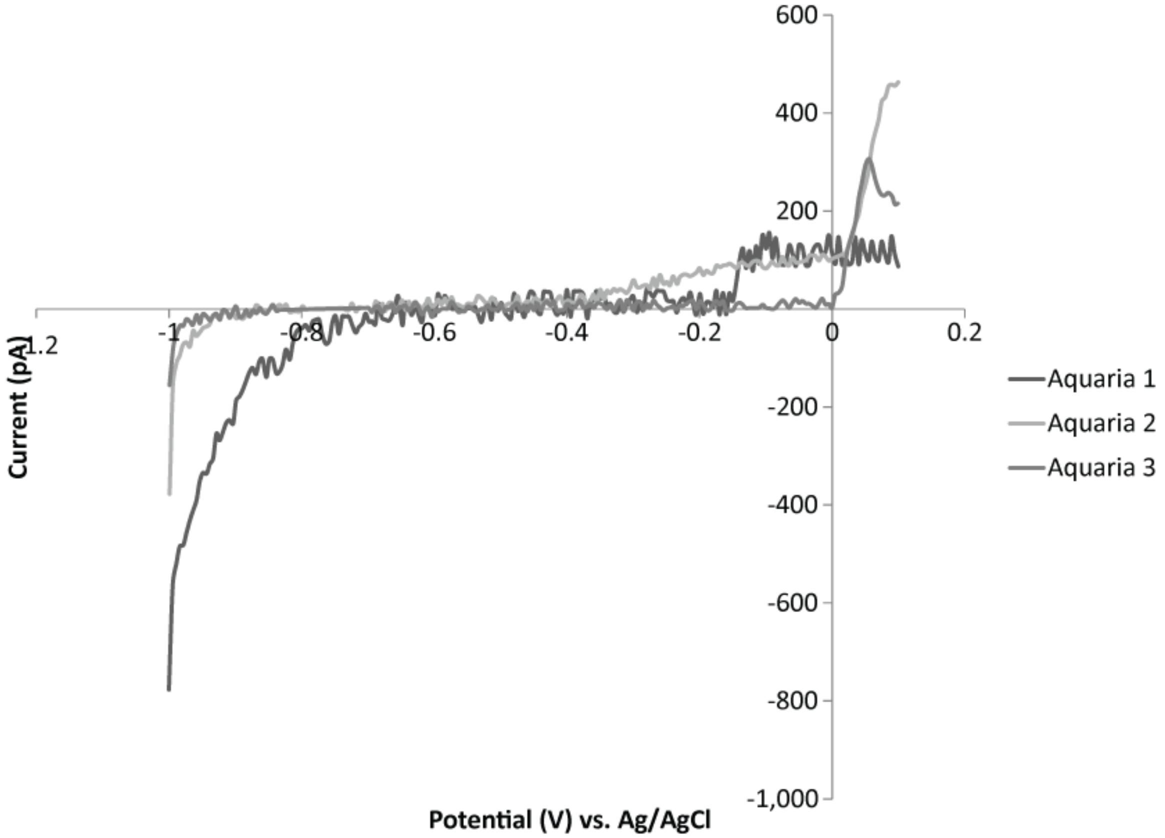

Figure S2. Linear Sweep Voltammetry (LSV) performed on replicate reactors constructed with newly sieved sediments from Catalina Harbor and UV treated Seawater. LSV run from -1.0 to 0.1 V at a scan rate of 0.3 mV/s.

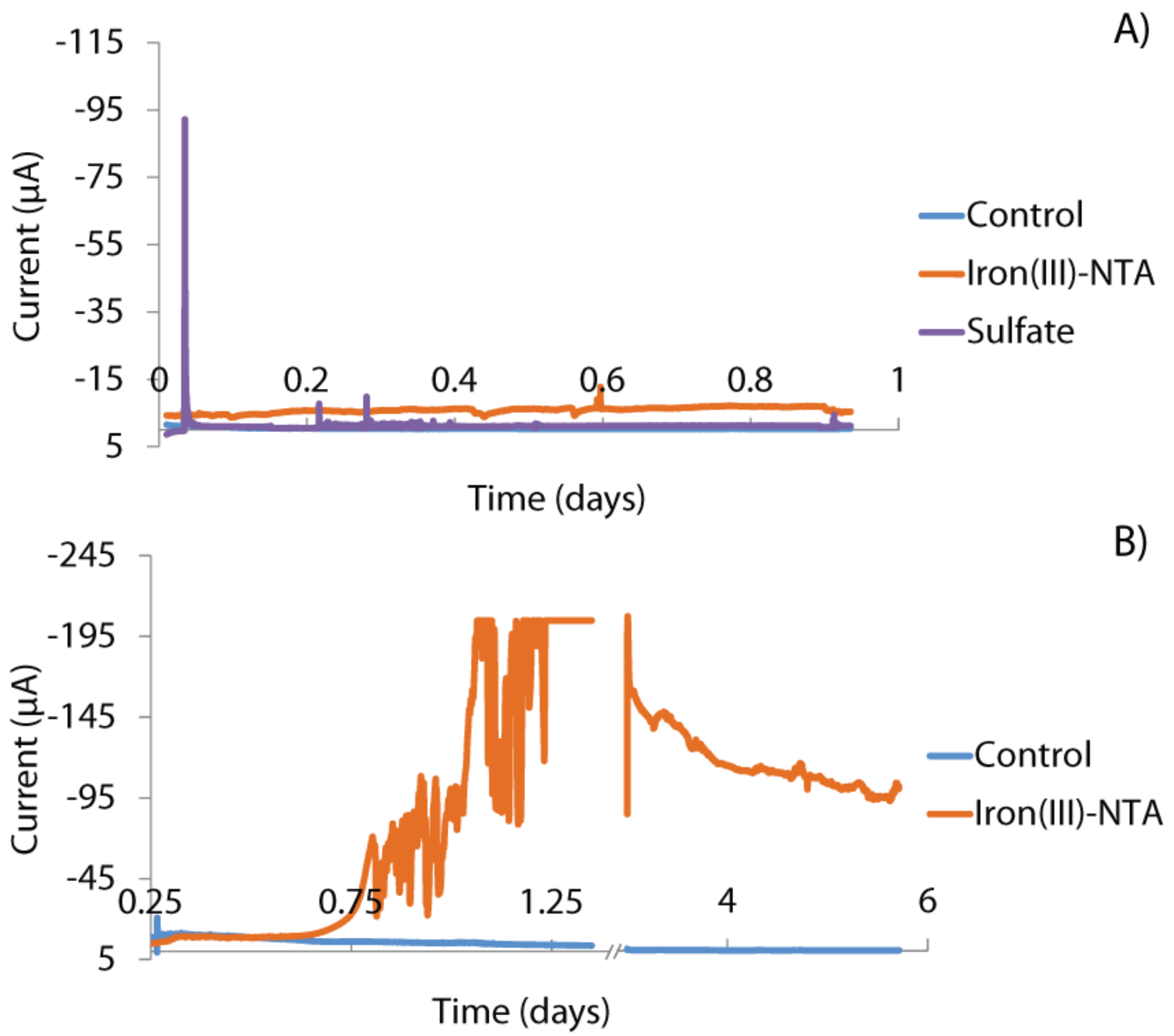

Figure S3. Chronoamperometry profiles for iron and sulfate amended sediment free bioreactors relative to control (sterile electrode). A) Represents early time points in the reactor while B) demonstrates current production after five consecutive feeds of 250 µM Ferric Iron NTA. In both of these profiles amendments were added to reactor at approximately time zero.

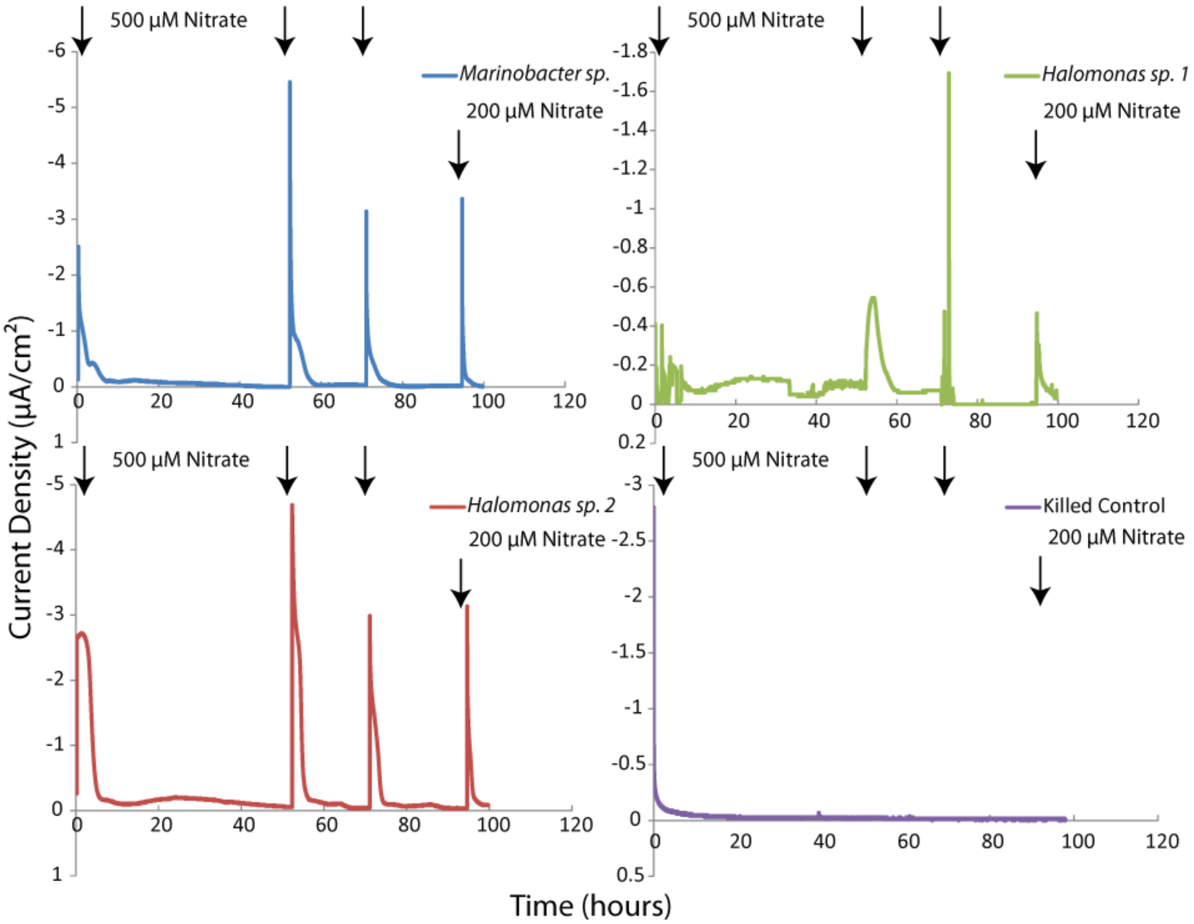

Figure S4. Selected chronoamperometry results for isolated culture from the *Marinobacter* and *Halomonas* genera of the *Gammaproteobacteria*. A killed control consisting of an autoclaved *Halomonas* culture was also tested. Periodic batch feeds of nitrate (first three 500 µM and last 200 µM) were added to stimulate current production as indicated by arrows.

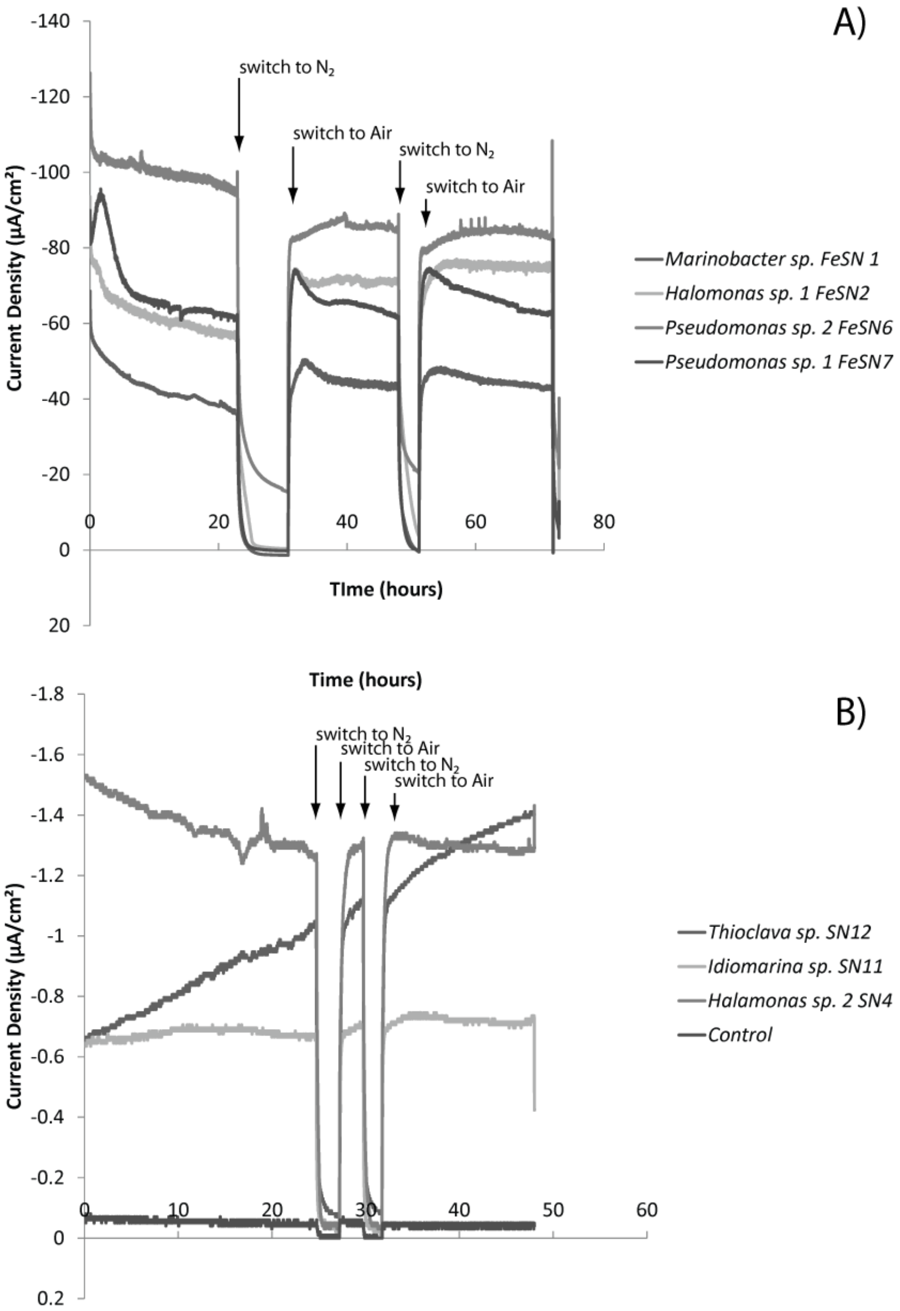

Figure S5. Chronoamperometry data for electrochemically active cultures at various stages of inoculation on either graphite (A) or ITO (B) electrodes. Current production in electrochemical cells linked to the presence of oxygen

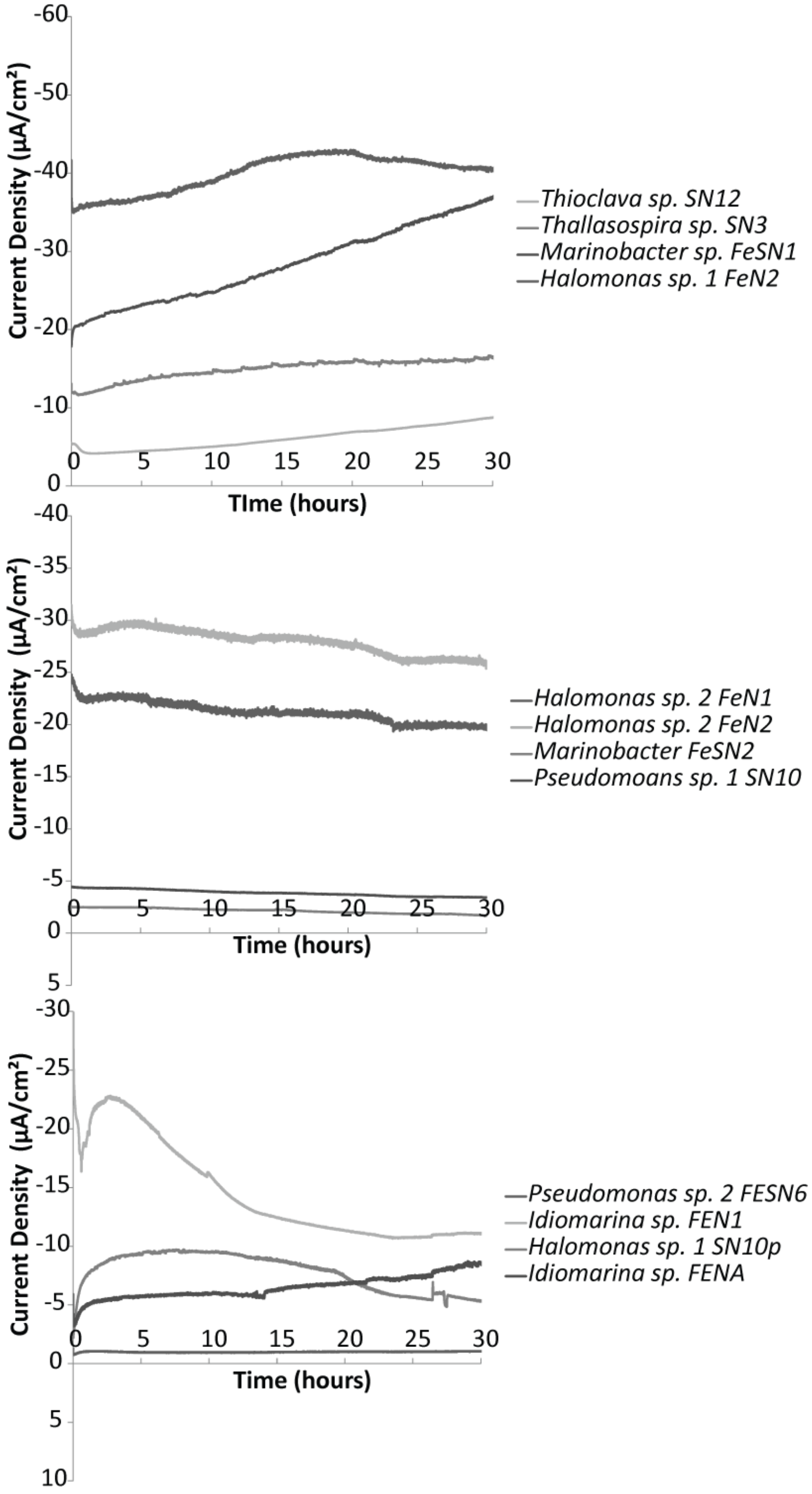

Figure S6. Chronoamperometry profiles for electrochemically active isolates grown in an electrochemical cell with oxygen as a terminal electron acceptor in the absence of organic carbon from two to four weeks. Electrodes were poised at -400 mV vs. Ag/AgCl.

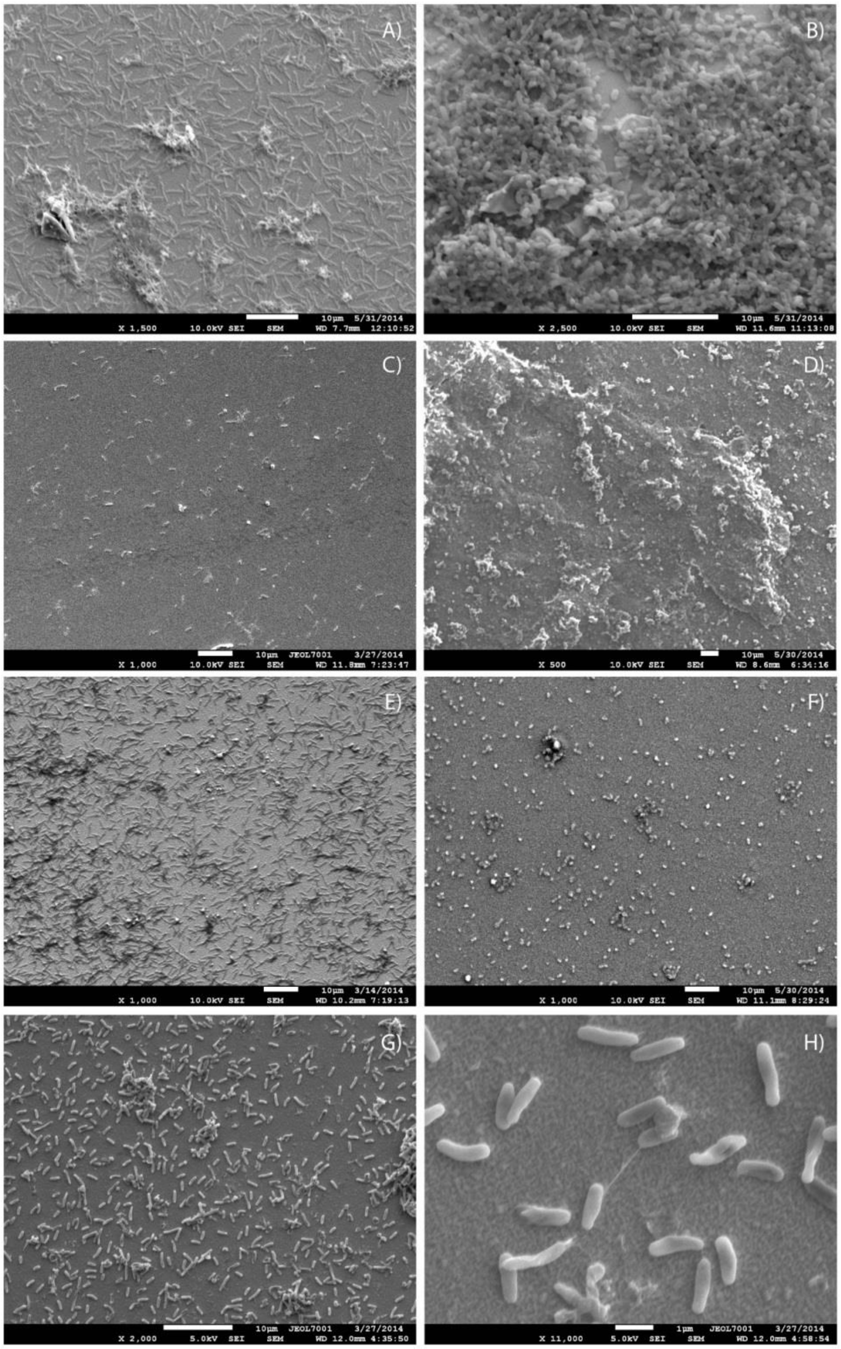

Figure S7. Representative Scanning Electron Micrographs of select glutaraldehyde fixed ITO electrodes. Electrodes taken from electrochemical cells for: A) *Pseudomons sp. 2* SN 5, B) *Halomonas sp. 2* FeSN 2, C) *Marinobacter sp*. FeSN 1, D) *Idiomarina sp.* SN 11, E) *Thalassospira sp.* SN 3, F) *Thioclava sp.* SN 12, G) *Halomonas sp. 1* FeN 2 and H)

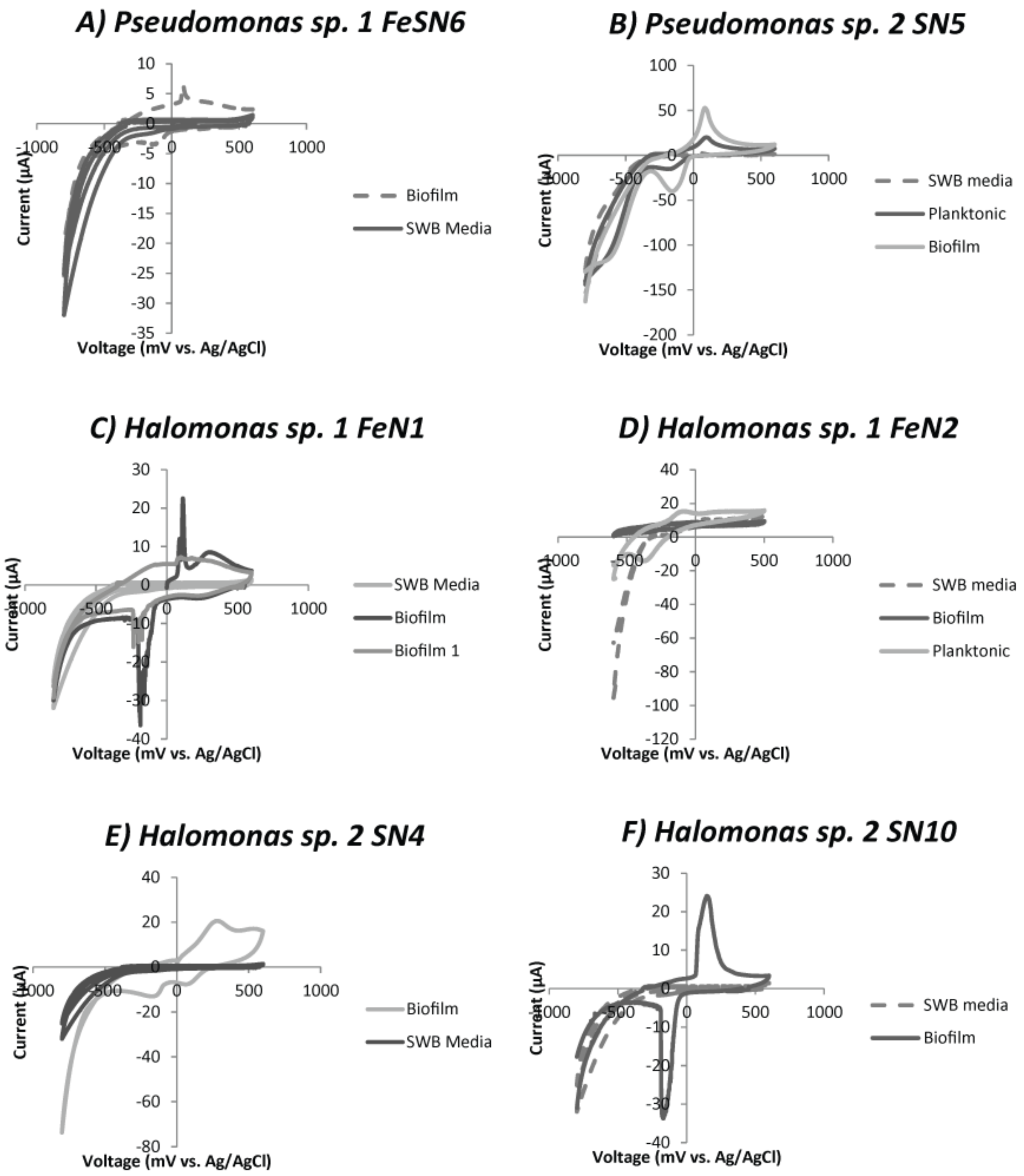
A) Pseudomonas sp. 1 FeSN6
10
5
0
-5
-10
-15
-20
-25
-30
-35
-1000
-500
0
500
1000
Current (µA)
Voltage (mV vs. Ag/AgCl)
Biofilm
SWB Media
B) Pseudomonas sp. 2 SN5
100
50
0
-50
-100
-150
-200
-1000
-500
0
500
1000
Current (µA)
Voltage (mV vs. Ag/AgCl)
SWB media
Planktonic
Biofilm
C) Halomonas sp. 1 FeN1
30
20
10
0
-20
-30
-40
-1000
-500
0
500
1000
Current (µA)
Voltage (mV vs. Ag/AgCl)
SWB Media
Biofilm
Biofilm 1
D) Halomonas sp. 1 FeN2
40
20
0
-20
-40
-60
-80
-100
-120
-1000
-500
0
500
1000
Current (µA)
Voltage (mV vs. Ag/AgCl)
SWB media
Biofilm
Planktonic
E) Halomonas sp. 2 SN4
40
20
0
-20
-40
-60
-80
-1000
-500
0
500
1000
Current (µA)
Voltage (mV vs. Ag/AgCl)
Biofilm
SWB Media
F) Halomonas sp. 2 SN10
30
20
10
0
-10
-20
-30
-40
-1000
-500
0
500
1000
Current (µA)
Voltage (mV vs. Ag/AgCl)
SWB media
Biofilm

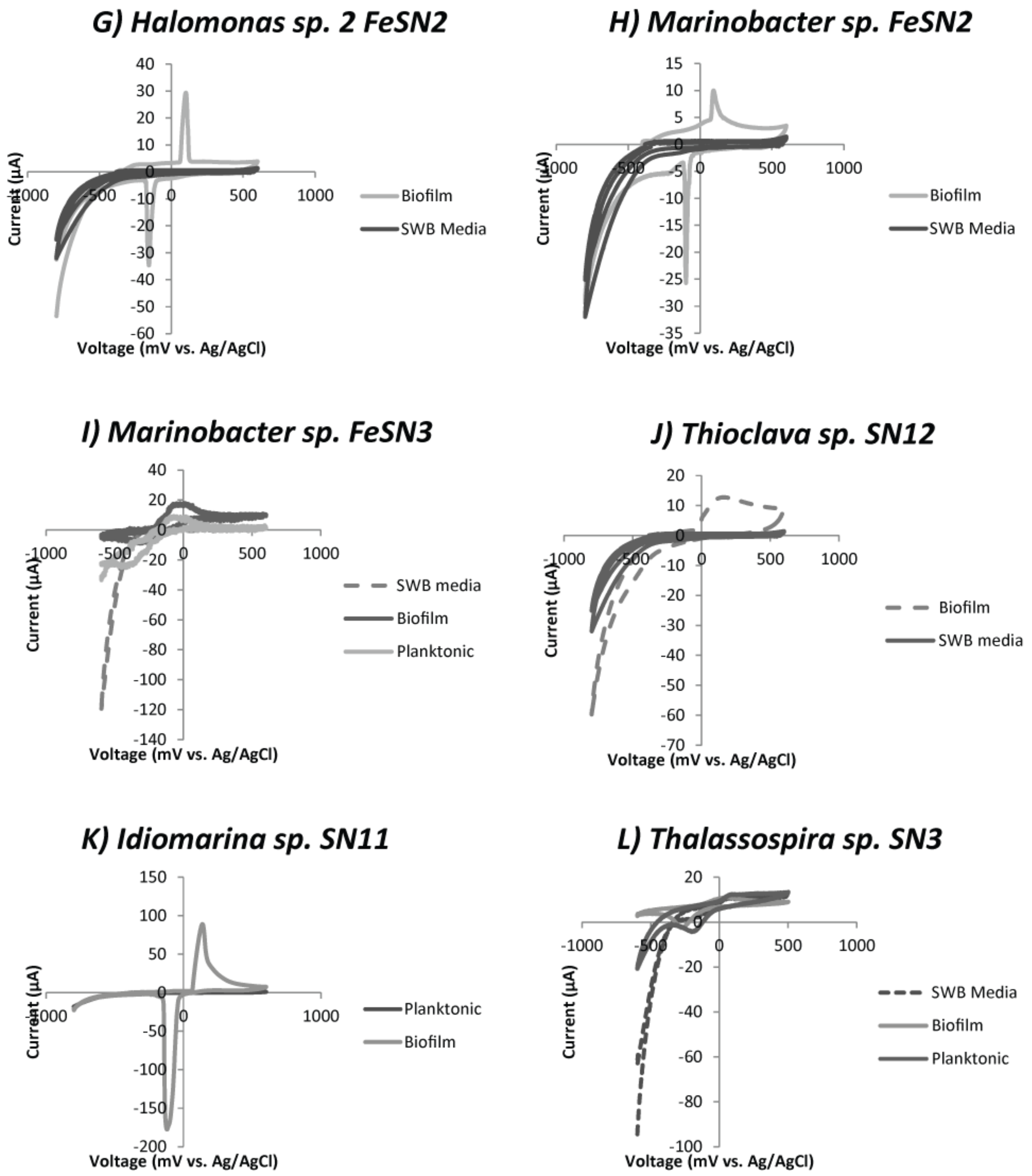

Figure S8. Representative cyclic voltammetry plots under non-turnover conditions for organism listed (A-L). Various strains and plots represented for the background media in a sterile cell (SWB) as well as planktonic (suspended cells in spent media) and biofilm (cell biomass attached to electrodes) associated biomass. Scans were run from 5-10 mV/s.
